# Forest gaps increase true bug diversity by recruiting open land species

**DOI:** 10.1007/s00442-023-05392-z

**Published:** 2023-06-04

**Authors:** Rafael Achury, Michael Staab, Nico Blüthgen, Wolfgang W. Weisser

**Affiliations:** 1 Terrestrial Ecology Research Group, Department of Life Science Systems, School of Life Sciences, Technische Universität München, Freising, 85354 Germany; 2 Ecological Networks Lab, Technische Universität Darmstadt, Darmstadt, Germany

**Keywords:** Gap dynamics, Dispersal ability, Insect body size, Deadwood amount, Feeding guild and stratum use

## Abstract

Forests canopy gaps play an important role in forest ecology by driving the forest mosaic cycle and creating conditions for rapid plant reproduction and growth. The availability of young plants, which represent resources for herbivores, and modified environmental conditions with greater availability of light and higher temperatures, promote the colonization of animals. Remarkably, the role of gaps on insect communities has received little attention and the source of insects colonizing gaps has not been studied comprehensively. Using a replicated full-factorial forest experiment (treatments: *Gap*; *Gap* + *Deadwood*; *Deadwood*; *Control*), we show that following gap creation, there is a rapid change in the true bug (Heteroptera) community structure, with an increase in species that are mainly recruited from open lands. Compared with closed-canopy treatments (*Deadwood* and *Control*), open canopy treatments (*Gap* and *Gap* + *Deadwood*) promoted an overall increase in species (+ 59.4%, estimated as number of species per plot) and individuals (+ 76.3%) of true bugs, mainly herbivores and species associated to herbaceous vegetation. Community composition also differed among treatments, and all 17 significant indicator species (out of 117 species in total) were associated with the open canopy treatments. Based on insect data collected in grasslands and forests over an 11-year period, we found that the species colonizing experimental gaps had greater body size and a greater preference for open vegetation. Our results indicate that animal communities that assemble following gap creation contain a high proportion of habitat generalists that not occurred in closed forests, contributing significantly to overall diversity in forest mosaics.

**Supplementary Information:**

The online version contains supplementary material available at 10.1007/s00442-023-05392-z.

## Introduction

Natural gap dynamics in forests are the consequence of natural tree mortality and disturbances, such as fires or storms, resulting in a change in microclimatic conditions on the forest floor toward higher light availability and lower humidity (Ritter et al. 2005; Feldmann et al. 2018). Gaps are often created by dying trees and thus associated with a high amount of available deadwood (Franklin et al. 2002), especially at early stages of forest succession (Hilmers et al. 2018). Forest gaps are generally colonized by more light-demanding plants species, resulting in a succession, where herbaceous plants are eventually replaced by woody vegetation and trees, until the gap is finally closed (Grime 2006; Kucbel et al. 2010), modifying the function and structure of forest ecosystems (Swanson et al. 2011; Zhu et al. 2014). These successional forests with different stages of regeneration, although different from the surrounding forests, are nevertheless important for biodiversity (Falster et al. 2017; Schall et al. 2018; Poorter et al. 2021).

Some animal species that colonize gaps may be species that live mainly in the adjacent closed forests (Staab et al. 2022), whereas other colonizing species might be gap specialists or species from open habitats, as is also true for most plants in the gap (Liira and Paal 2013). For example, bird communities inhabiting early successional stages of forest are primarily obligate granivores or insectivores, compared with birds occupying closed forest which are specialist feeders, particularly frugivores (Bowman et al. 1990). Animal community assembly in forest gaps is affected by plant successional patterns, with most studies focusing on vertebrates (Gitzen and West 2002; Moorman et al. 2007; Pollock et al. 2020). For insects, there are fewer studies (Richards and Windsor 2007; Eckerter et al. 2021; Staab et al. 2022), and there is some evidence for butterflies that newly created forest gaps are first colonized by species with better abilities for dispersal (Viljur and Teder 2018). Gaps can also affect species not specialized on herbaceous vegetation. For deadwood-colonizing species, in particular saproxylic beetles, it has been shown that light-exposed deadwood is preferentially colonized by species that also largely occur in sun-exposed deadwood, i.e., in gaps (Seibold et al. 2016; 2018). For non-saproxylic species, however, there is less information on whether the species colonizing gaps are species that generally prefer open habitats, or if these newly created areas due to forest management promote differences in body size (Staab et al. 2023).

True bugs (Hemiptera: Heteroptera) are a suitable group to study community assembly in forest gaps, because they are a highly diverse group of insects inhabiting a great number of environments including different strata within forests (Sobek et al. 2009; Leidinger et al. 2019). They also respond to changes in light intensity (Gossner 2009). Moreover, Heteroptera play several roles within the ecosystems acting as herbivores and carnivores, both specialist and generalist. Thus, as a result of their high degree of host-plant specialization and based on their feeding habits as sucking insects, true bugs might respond to environmental gradients such as those presented by gaps (Knuff et al. 2020). Heteroptera may thereby not only respond to the vegetation in the gap, but also to the presence of deadwood where they would act as predators or fungivores (Seibold et al. 2014).

One particular challenge in understanding the mechanisms underlying gap dynamics of saproxylic, but also non-saproxylic species, is that gap creation in natural forests is invariably correlated with deadwood formation leading to concomitant changes in both, deadwood availability, and light and microclimatic conditions (Feldmann et al. 2018). Deadwood not only influences resource availability for saproxylic species, but can also affect plant succession in gaps, and the creation of microhabitats for animals, e.g., in fallen tree crowns (Bouget and Duelli 2004). To disentangle the role of two important factors associated with forest management, the change of abiotic conditions by opening the canopy and the availability of deadwood depending on logging, large-scale experiments are required (Weisser et al. 2023), but difficult to execute and maintain. Managed forests offer the opportunity to separate gap creation from deadwood enrichment, as gaps are also created by forestry practices such as felling of individual trees (Schall et al. 2018). Although a large number of studies on gap dynamics of natural forests have been developed (Yamamoto 2000; Asner et al. 2013), experimental investigations of gap dynamics in managed forests are still limited (Kozel et al. 2021).

In this study, we report some of the earliest results of a forest gap experiment (see Staab et al. 2022), where gap formation and deadwood provisioning were experimentally manipulated independently, in a 2 × 2 full-factorial design. Specifically, we used true bug communities sampled for two consecutive years to test the following hypotheses: (1) gap treatments (i.e., with open canopy) will harbor a higher number of individuals and species of true bugs compared with closed-canopy treatments; (2) the community composition of true bugs in gaps will be distinct and not just a random subset of the species pool in surrounding forest; and (3) species colonizing gaps are species that generally prefer open habitats, such as grasslands (Supplementary material: Fig. S1).

## Materials and methods

### Study system

**Fig. 1:**
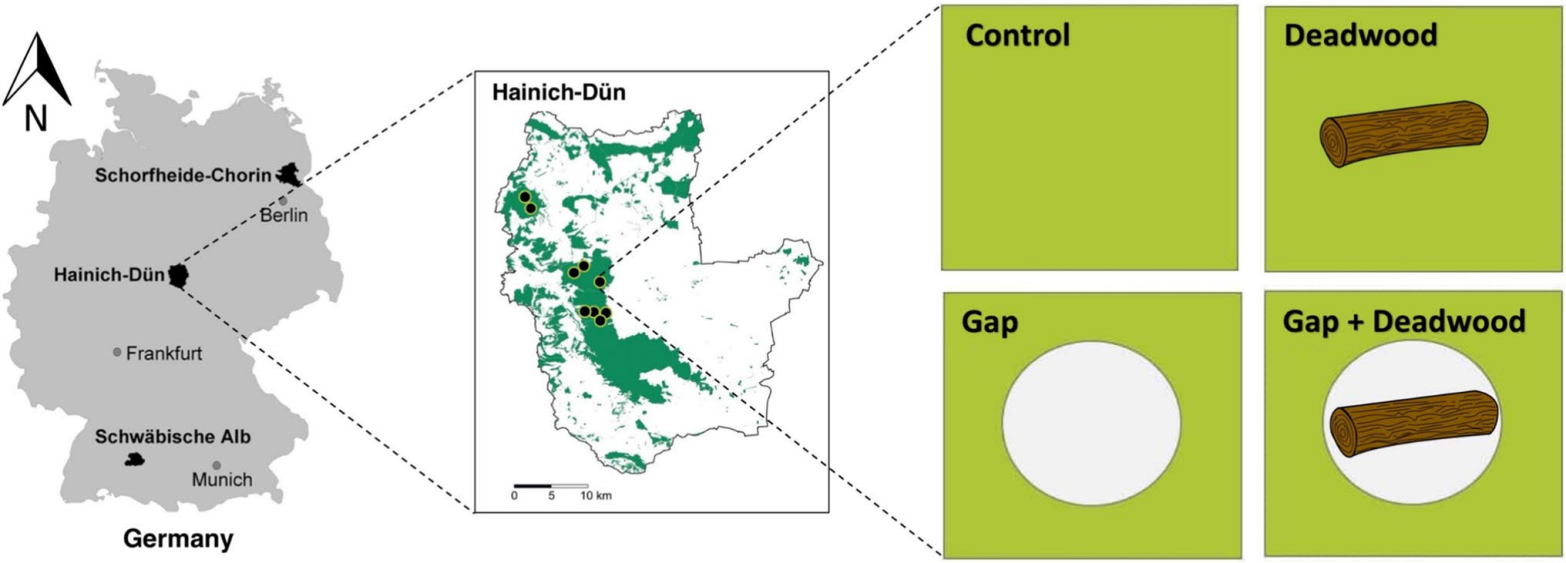
Map of the study area representing the nine sites sampled in Hainich-Dün, indicated by black dots in the central panel. A full-factorial experiment was created in winter 2019 and early spring 2020 in forested areas (depicted in green) in which four treatments (i.e., *Control*, *Deadwood*, *Gap,* and *Gap* + *Deadwood*) were established to disentangle two important factors related with forest management: gap creation and deadwood availability. In total, 36 plots were established (9 sites × 4 treatments)

This study was conducted within the Biodiversity Exploratories framework (www.biodiversity-exploratories.de), a large-scale and long-term research project to investigate the impacts of land use on biodiversity and associated ecosystem functions and services (Fischer et al. 2010). The study area is located in the region of Hainich-Dün, northwestern of Thuringia in central Germany (Fig. 1). The Hainich-Dün region includes the National park Hainich and its surroundings, the forest landscape is composed mainly by managed forests with European beech (*Fagus sylvatica*) as main tree species. With approximately 16,000 ha, the study area covers 50 km direction north–south and 42 km direction east–west (50°56′14″–51°22′43″N, 10°10′24″–10°46′45″E), and the elevation ranges from 258 to 550 m above sea level. The region has a typical temperate climate, with an average annual temperature of 6–7 ℃ and a mean annual precipitation of 500–800 mm.

### The forest gap experiment

In winter 2019 and early spring 2020, a multi-site full-factorial FOrest gap eXperiment (FOX) was set up to investigate the effects of manipulations of forest openness (gap) and deadwood amount, in 9 beech forest stands out of the 50 forest sites associated with the Hainich region in the Biodiversity Exploratories project (Fischer et al. 2010). These nine sites reflect the dominant tree species with five even-aged beech sites and four uneven-aged beech sites. The experiment (Staab et al. 2022) investigates the effects of creating gaps with and without deadwood and it is based on four treatments per site (Fig. 1):

*Control:* this corresponds to a regular plot sampled within the frame of the Biodiversity Exploratories (Fischer et al. 2010), in which no gap was created and where no additional deadwood was placed.

*Gap:* a gap was created with a diameter of around 30 m (mean = 34.0 m; SD ± 1.24 m, range 30–37 m) and all cut trees were removed.

*Gap* + *Deadwood:* an identical sized-gap was created, but half of the deadwood was left in the plot. Each felled tree was cut into four equally sized parts, of which two were placed in the plot.

*Deadwood:* in an unmodified forest plot adjacent to the other plots within a site, the additional two equally sized parts of the trees felled in the *Gap* + *Deadwood* treatment were added to the plot.

All plots were spatially arranged to minimize variation in topography and exposition, keeping a minimum distance of 100 m among treatments. In total, 562 trees were cut to create the gaps in Hainich and the logs were moved among plots using man power and heavy machinery. Each control plot is equipped with a weather station, which records air temperature (measured at 200 cm above ground, accuracy 0.01 °C) every 10 min, and precipitation, which is estimated based on radar sensors (RADOLAN) by the German Weather Service (accuracy 0.1 mm/h). In 2020 and 2021, the years of collection, average annual temperature for the nine sites was 8.2 °C, and the mean precipitation was 534 mm.

### True bug sampling

In each of the 36 experimental plots (9 sites × 4 treatments), two flight-interception traps were installed on a wooden frame 1.5 m above ground to capture flying insects (Supplementary material: Fig. S2). Traps consisted of two transparent plastic cross panels (40 × 60 cm) with two funnels attached at the bottom and at the top in which sampling jars were filled with a solution of CuSO_4_ (3%) and a drop of detergent to reduce surface tension. Window traps, which provide an estimate of true bug activity for each species by collecting the number of individuals that fly into both jars for each monthly sample period, are an effective method for sampling insect forest communities (Knuff et al. 2019). Although this measure of activity may be biased due to differences in foraging pattern, stratum use, and wind development among species (Gossner et al. 2015), it is relatively unbiased and widely used for comparisons among areas for flying insect communities. Traps were installed in 2020 from April to July, while, in 2021, they were exposed from March to July, emptied monthly, and all specimens preserved in 100% ethanol in the laboratory of the Terrestrial Ecology group at the Technical University of Munich (Germany). In the laboratory, true bugs were sorted out from the samples, and all adult individuals identified to species level by a contracted taxonomist (see acknowledgments). In 2020, in each of the 36 plots, we took five measurements related to vegetation, including the percentage of ground coverage, the average, and maximum height. These measurements were taken from five subplots of 1 m^2^, with one located at the center of the plot and the other four placed at each cardinal point, spaced 9 m apart (Staab et al. 2022).

For all identified species we compiled information from the literature on feeding guild, stratum use, dispersal ability, and body size (Gossner et al. 2015; Seibold et al. 2019). True bugs were assigned to one of three main feeding guilds based on their known main food resource as adults (i.e., carnivores, herbivores, and omnivores); while for stratum use, they were divided into four groups on the basis of the main vegetation layer (vertical stratum) in which the adults of the species are usually found (i.e., ground, herb, shrub/tree, or intermediate). For the majority of arthropod species, we do not have information regarding their usual distance of dispersion; therefore, dispersal ability was based on the level of wing dimorphism between males and females, description of flying abilities and/or dispersal strategies, and can take values between 0 (lowest dispersal ability) and 1 (highest dispersal ability), with the highest value of 1 for species with fully developed wings in both sexes; 0.75 for predominantly macropterous species; 0.5 for equally brachypterous and macropterous species; 0.25 for predominantly brachypterous species; and 0 for always brachypterous species (for further details on wing measurements and dispersal ability calculation, see Gossner et al. 2015; Simons et al. 2016). Wing dimorphism between males and females is an important indicator of a species' dispersal capacity, as mainly winged or macropterous individuals are responsible for escaping deteriorated habitats and dispersing into new ones (Zera and Denno 1997). Finally, body size, a trait that has been demonstrated to exhibit variability in response to disturbances or forest management interventions (Ribera et al. 2001), was determined by calculating the average body length in mm for both males and females (see Gossner et al. 2015 for detailed description).

### Statistical analyses

All statistical tests were conducted in R 4.2.1 (www.r-project.org), and some graphs were produced with the *ggplot2* package, version 3.3.6 (Wickham 2016).

#### Diversity of true bug species

Number of species and abundances of heteropterans were pooled for each plot and over years. Only adult specimens were used in the analyses. First, to assess how species density (number of species per plot) (sensu Gotelli and Ellison 2013) and number of individuals per plot varied with treatments, we used generalized linear mixed models (GLMMs) with negative binomial errors (using the *glmmTMB* package, version 1.1.4; Brooks et al. 2017). Separate models were fit with feeding guild (three levels) and stratum use (four levels) as additional explanatory variables, and their corresponding interaction with the treatments. In all models, “Site” was included as a random intercept to account for potential non-independence among treatments within the same site. Pairwise contrasts among treatments were calculated post hoc using Bonferroni correction to account for multiple comparisons (*emmeans* package, version 1.8.0; Lenth 2022). To test whether treatment effects on species density are mediated by abundance (i.e., more-individual hypothesis, Srivastava and Lawton 1998) or whether they are true effects on species density, we calculated an additional model in which we included “abundance” as a fix effect into the model for species density. In this case, abundance was fitted first, and an analysis of deviance with sequential sum of squares (type I SS) was applied (*car* package, version 3.1–0; Fox and Weisberg 2019) to separate the mediating effect of species abundance in relation with the treatments. The fit and validation of all the models were evaluated with the *DHARMa* package (version 0.4.5; Hartig 2022).

In addition to species density, we also analyzed total true bug diversity per treatment using the rarefaction/extrapolation framework of Chao et al. (2014) in the *iNEXT* package (version 3.0.0; Hsieh et al. 2022). Species diversity was estimated based on sample coverage rather than sample size, because the latter might be insufficient to characterized richer communities (Chao and Jost 2012). Three measurements of species diversity based on Hill numbers were calculated: (a) Species richness (*q* = 0) weights all species equally and thus emphasizes rare species, (b) Shannon entropy (*q* = 1) weighs all species by their frequency, without favoring rare or dominant species, and (c) Simpson diversity (*q* = 2) assigns most weight to dominant species (Jost 2006). Based on 1000 bootstraps, we calculated confidence intervals (CI = 95%) for the three measures of species diversity and treatments were compared at the lowest sample coverage among them (96.3%) (Chao et al. 2014), allowing for a standardized comparison of true bug assemblage diversity among treatments despite differences in their abundance.

#### Species composition and indicator analysis

To compare true bugs’ community composition among treatments, a non-metric multidimensional scaling (NMDS) ordination with two dimensions was calculated, using the metaMDS function (*vegan* package, version 2.6-2; Oksanen et al. 2022) with Bray–Curtis dissimilarity and 9999 permutations. Abundances were standardized (Wisconsin-double) and sqrt-transformed before calculating dissimilarity. To test for differences in composition among treatments, a multivariate analysis of variance (PERMANOVA) with 9999 permutations, and subsequently pairwise contrasts were conducted using the adonis2 and the pairwise.adonis functions from the *vegan* package.

In addition, we measured the degree of association of true bug species with each treatment using indicator species analysis (Dufrêne and Legendre 1997) with the *indicspecies* package (version 1.7.12; de Cáceres and Legendre 2009). This technique generates indicator values ranging from 0 (no indicator value) to 1 (perfect indicator to be always present and exclusive to a particular treatment). The indicator value is calculated as the product of two quantities, A and B, where A is a probability of a species as an indicator of a treatment (or combination of treatments, e.g., *Control* and *Deadwood*), and B is a measure of how frequently the species is found at the sites in each of the treatments (de Cáceres et al. 2010). “Indicator values” are assigned to each species in each treatment, and we assessed the statistical significance of the maximum indicator value for each species using a Monte Carlo test in which the abundance of species between treatments was randomized (9999 permutations).

#### Species colonizing gaps

Literature data on grassland or forest specialization are rare for Heteroptera, and when available, it is just for a small subset of species providing concise descriptions of the environment used by these species, without quantitative data to estimate preference levels (Deckert and Wachmann 2020). Thus, we calculated true bugs “*open-vegetation preference*” (following Frank et al. 2017) for 114 species (117 species in total, but three species were new for the Biodiversity Exploratories project and had no previous data, and thus, they were not included in the analyses: *Leptoglossus occidentalis*, *Scoloposcelis pulchella*, and *Tingis pilosa*) as a measure of their relative occurrence in open habitats. We did this using data from 2008 to 2018 collected in forest (by means of flight-interception traps) and grassland plots (using sweep netting) within the Biodiversity Exploratories project (Seibold et al. 2019), to estimate for each species the percentage of the number of individuals collected in grasslands among all plots (150 forest plots + 150 grassland plots) (i.e., *“open-vegetation preference”*). This percentage allowed us to obtain a comparable metric for the species recorded in our study based on grassland specialization (i.e., occurring primarily in grasslands). This information of “*open vegetation preference*” was then compared, using logistic regression (binomial errors and a logit link function) with the percentage of individuals captured in 2020 and 2021 in treatments with open canopy (i.e., *Gap*, and *Gap* + *Deadwood*) among all experimental treatments (*Control*, *Deadwood*, *Gap*, and *Gap* + *Deadwood*) (i.e., *“gap preference”*). It is important to note that this “*open-vegetation preference*” describes preference for grasslands, as this is the open land-cover type sampled in Biodiversity Exploratories (Fischer et al. 2010). Our sampling does not include other habitats, such as hedges, farmlands, or wetlands, and, therefore, should not be considered as an absolute, but rather a relative, measure of species specialization to open-vegetation habitats (Penone et al. 2019).

Similarly, we took advantage of additional previous sampling in the forest canopy (Biodiversity Exploratories project) to investigate if species closely associated with this forest layer were colonizing the newly created treatments with open canopy (i.e., *Gap*, and *Gap* + *Deadwood*). Thus, with the information gathered from window traps displayed contemporaneously from 2008 to 2012 (Gossner et al. 2013) both in the canopy and the understory (abundance per species was positively correlated: Pearson’s *r* = 0.8; *P* < 0.001), we calculated the “*canopy preference*” for each species based on the percentage of the number of individuals collected in traps located in the canopy among all traps (canopy + understory). We use a logistic regression to test if the “*gap preference*”, i.e., percentage of individuals captured in 2020 and 2021 in treatments with open canopy (i.e., *Gap*, and *Gap* + *Deadwood*) among all experimental treatments (*Control*, *Deadwood*, *Gap*, and *Gap* + *Deadwood*) was related with the “*canopy preference*” calculated for the true bug species. We ran this analysis with binomial error structure and the logit link function.

Finally, we investigated if the species colonizing the treatments with open canopy (*Gap* and *Gap* + *Deadwood*) were characterized by higher dispersal ability and changes in body size compared with the species inhabiting closed-canopy treatments. For these analyses, we calculated the community-weighted means (CWM, weighted by the relative abundance of each species) for dispersal ability and body size in each community inhabiting the treatments and compared them using a linear mixed-effect model (LMM), where “Site” was used as a random intercept (*glmmTMB* package, version 1.1.4; Brooks et al. 2017).

## Results

### True bug community sampled

We recorded 2556 adult true bug individuals from 117 species and 85 genera in the window traps displayed during 2020 and 2021 (mean: 71 ± 14.3 individuals/plot SE, Supplementary material: Table S1). The most abundant species was *Palomena prasina* with 803 individuals, representing 31.4% of the total individuals collected, followed by *Dolycoris baccarum* (381 individuals: 14.9%) and *Psallus varians* (255 individuals: 10.0%). The remaining 114 species each contributed less than 6% of individuals, and 61 species were either singletons (40 species: 34.2%) or doubletons (21 species: 17.9%).

### Effect of gap and deadwood treatments on true bug abundance and diversity

**Fig. 2:**
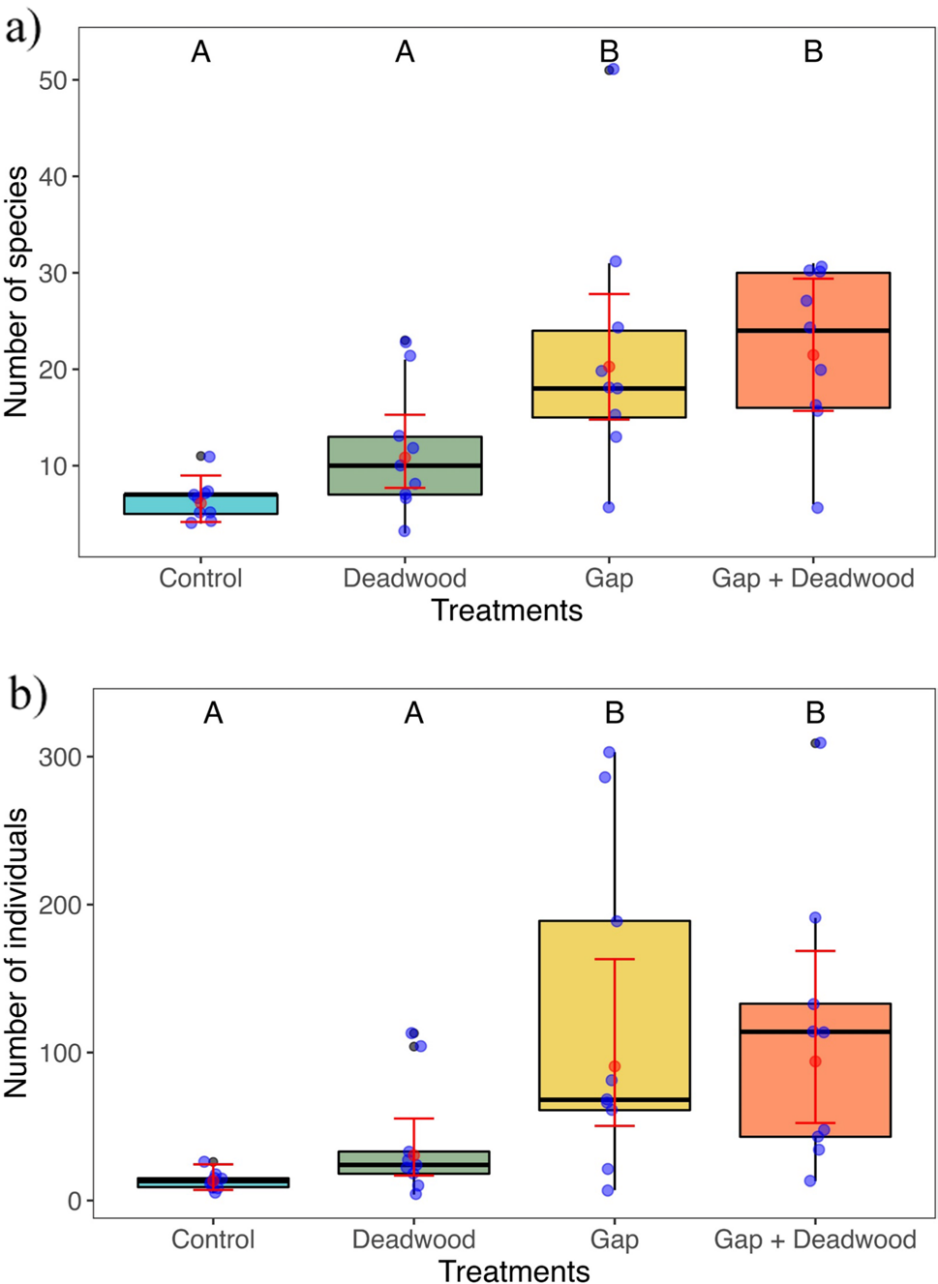
Differences in **a** number of species and **b** individuals per plot among experimental treatments. Blue dots are raw data, while the red dot is the average estimate by the negative binomial generalized mixed model (± SE). Different letters indicate statistical significance at *P* < 0.05. Pairwise contrasts are reported in Table S2

Treatments with open canopies (*Gap* and *Gap* + *Deadwood*) had higher species density (Fig. 2a) and abundance per plot (Fig. 2b) compared with closed-canopy treatments (pairwise contrasts, *P* < 0.05, Supplementary material: Table S2). These results are driven by a substantial increase in the number of herbivores in the open canopy treatments (*P* < 0.05, Supplementary material: Fig. S3), while the carnivores and omnivores remained relatively constant across treatments. Although we did not find differences in vegetation ground coverage (*F*_(3, 32)_ = 0.702; *P* = 0.558) and height among treatments (average height: *F*_(3, 32)_ = 0.133; *P* = 0.940, and maximum height: *F*_(3, 32)_ = 0.764; *P* = 0.522), there were, in open canopy treatments, a significantly higher number of true bugs associated with the herbaceous stratum relative to those species with ground or shrub/tree associations (pairwise contrasts, *P* < 0.05, Supplementary material: Fig. S4).

When abundance per plot was included in the model of species density for all species, this variable was significantly related to the number of species per plot (i.e., species density) (*χ*^2^ = 40.145; df = 1; *P* < 0.001). Nevertheless, even after accounting for abundance, the treatment effect remained significant (*χ*^2^ = 22.68, df = 3; *P* < 0.001), and all open canopy treatments (i.e., *Gap* and *Gap* + *Deadwood*) harbored more species than the *Control* treatment, except for the *Deadwood* treatment which did not differ from the other three treatments (pairwise contrasts, *P* < 0.05, Supplementary material: Table S3, Fig. S5).

Yearly abundances per species were correlated among years (2020 vs. 2021: Pearson’s *r* = 0.68; Supplementary material: Fig. S6), and we observed a temporal increase in species density across all treatments, with higher numbers recorded in 2021 compared to 2020 (pairwise contrasts, *P* < 0.05, Supplementary material: Fig. S7a). Furthermore, the relationship among treatments remained consistent over the 2 years, with open canopy treatments harboring a higher species density than closed-canopy treatments. With respect to abundance, there was no difference between years in the number of individuals collected within the same treatment, and the number of individuals was higher for the open canopy treatments in 2020. However, in 2021, the abundance of true bugs in *Deadwood* increased, and harbored intermediate number of individuals between open canopy treatments and the *Control* (pairwise contrasts, *P* < 0.05, Supplementary material: Fig. S7b).

**Fig. 3:**
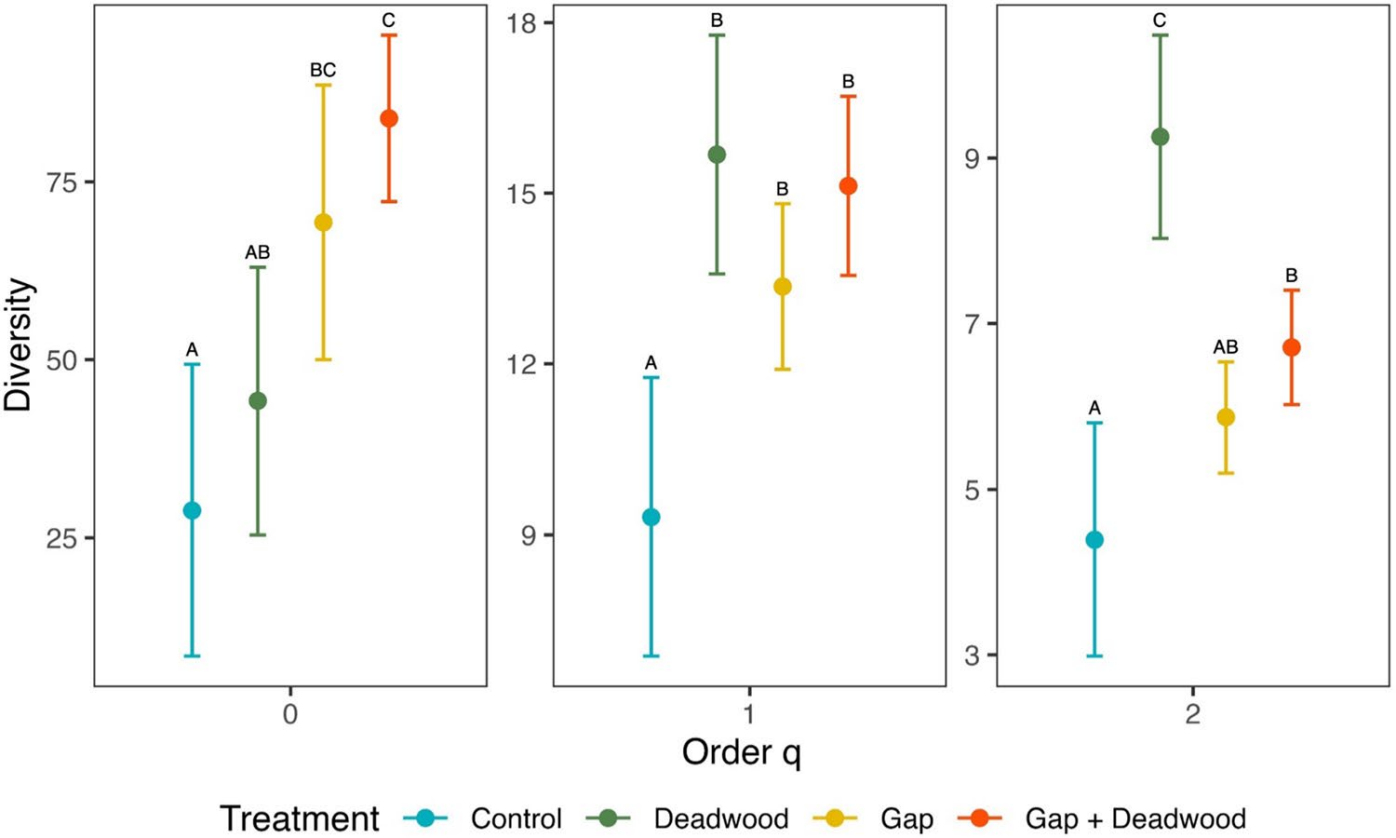
Coverage-based rarefaction/extrapolation graph with 95% confidence intervals (based on a bootstrap method with 1000 replications) comparing true bug species diversity (Hill numbers = q0: Species richness; q1: Shannon diversity; q2: Simpson diversity) in four experimental treatments in the region of Hainich-Dün. Different letters indicate statistical significance at* P* < 0.05

When samples were standardized using sample completeness (96.3%), the coverage-based rarefaction and extrapolation revealed that total species richness (*q* = 0) was lower in the *Control* (29 spp, CI ± 20.5) compared to treatments with open canopy (*Gap*: 69 spp, CI ± 19.3; *Gap* + *Deadwood*: 84 spp, CI ± 11.7), while the *Deadwood* treatment was significantly lower than the *Gap* + *Deadwood* treatment, but not significantly different from the *Control* and *Gap* treatments (44 spp, CI ± 18.8) (Fig. 3). The diversity of common species (*q* = 1) was lowest in the *Control* compared with the other three treatments (Fig. 3), which had at least 30% more species (*Deadwood* =  + 43.75.%, *Gap* =  + 30.77% and *Gap* + *Deadwood* =  + 40%). The diversity of dominant species (*q* = 2) was significantly higher in *Deadwood* (9 spp, CI ± 1.2), and lower in *Control* (4 spp, CI ± 1.4) with intermediate values for *Gap* (6 spp, CI ± 0.7) and *Gap* + *Deadwood* (7 spp, CI ± 0.7) (Fig. 3).

### Effect of gap and deadwood treatments on true bug community composition

**Fig. 4:**
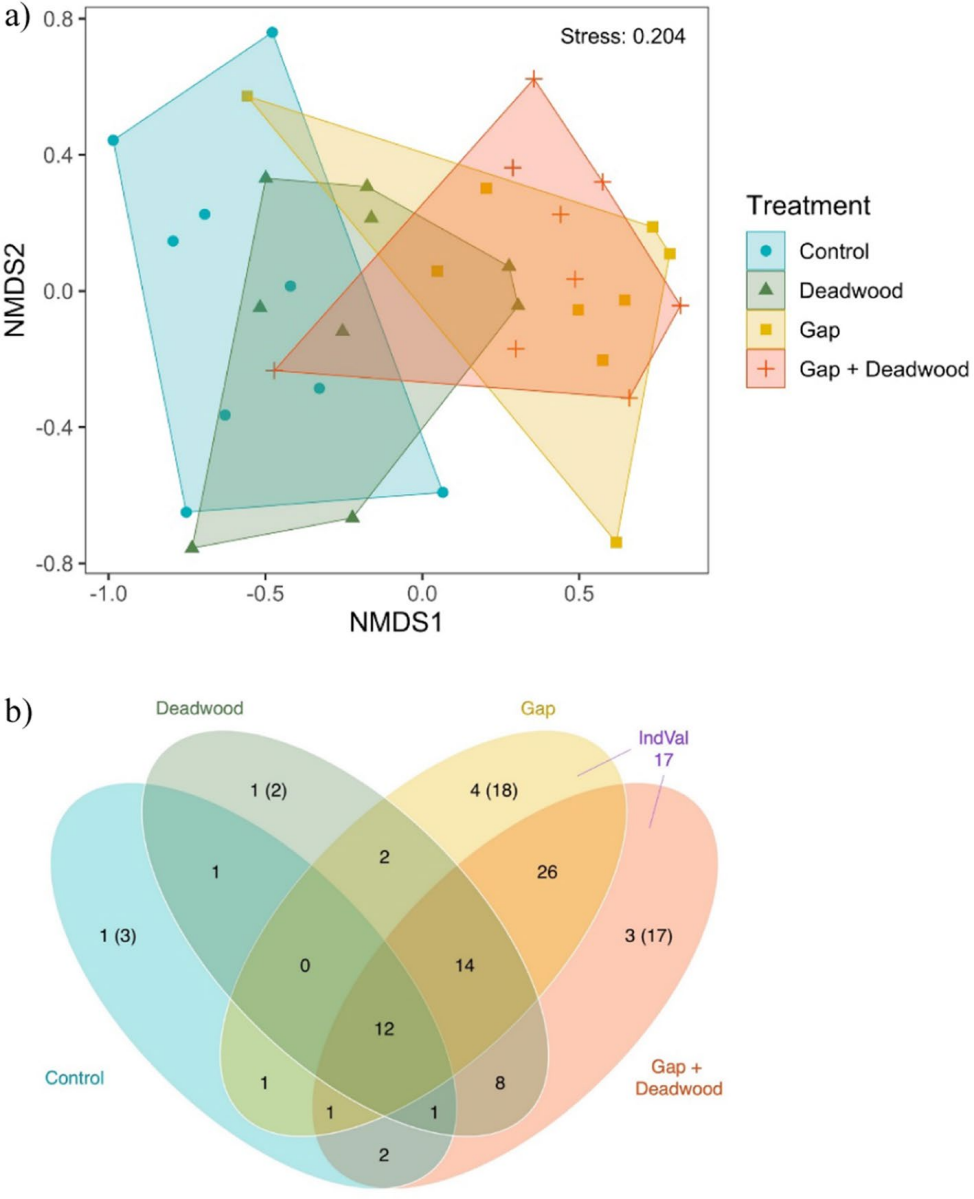
**a** Two-dimensional NMDS plot with Bray–Curtis distance based on abundances for treatments within FOX experiment in Hainich 2020 and 2021. **b** Number of species detected in each of the four experimental treatments. The Venn diagram shows the species shared among treatments (intersection of circles) and the singletons (number in parenthesis). The label “IndVal” refers to the number of species significantly associated with the combination of treatments with open canopies (*Gap* and *Gap* + *Deadwood*)

True bug community composition differed significantly between treatments, with two NMDS axes required for sufficient community representations (PERMANOVA: *F*_(3,32)_ = 3.250, *P* = 0.001) (Fig. 4a). Similar to what was observed in the analyses of abundance and species richness, species composition was largely separated into two main groups, treatments with open canopies (i.e., *Gap* and *Gap* + *Deadwood*), which also shared the highest proportion of species (22%, Fig. 4b), and the *Control* treatment (pairwise contrasts, *P* < 0.05; Supplementary material: Table S4). In contrast, true bug community composition of the *Deadwood* treatment overlapped non-significantly with the other three treatments (Fig. 4a and Supplementary material: Table S4).

### Association of particular true bug species with gap and deadwood treatments

**Fig. 5:**
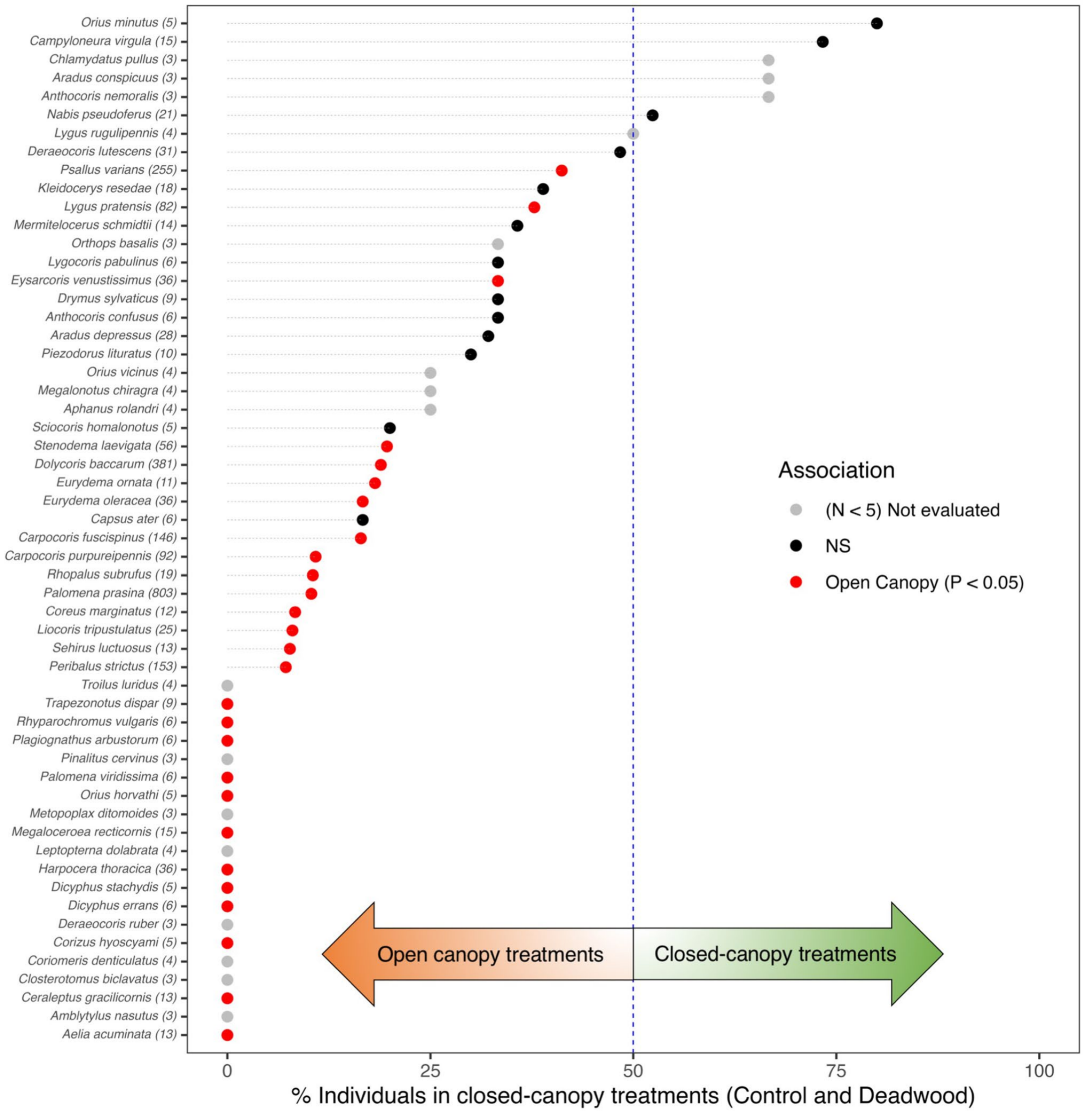
Distribution of individuals of different true bug species among open canopy treatments (*Gap* and *Gap* + *Deadwood*) and closed-canopy treatments (*Control* and *Deadwood*). A significant association with closed canopy/open canopy was tested using the Chi-square tests, separately for each species. Red dots: species with significant association (*P* < 0.05). Black dots: species not significant (NS) but with sufficient individuals (*N* ≥ 5) to be tested for association. Gray dots: species with < 5 individuals and not tested. Singletons and doubletons were excluded to improve readability. Number in parenthesis next to scientific name represents number of individuals

Twelve species were found in all four treatments, while 49 species were captured in just one of the treatments (Supplementary material: Table S1). *Palomena prasina* was the most abundant species in three treatments: *Deadwood* (74/355 individuals: 20.8%), *Gap* (394/1082: 36.4%), and *Gap* + *Deadwood* (326/999: 32.6%). In contrast, *P. varians* dominated the *Control* treatment (54/120: 45.0%) (Supplementary material: Table S1). Seventeen species were significant indicators for the combination of treatments *Gap* and *Gap* + *Deadwood* (Fig. 4b), with indicator values ranging from 0.58 to 0.33 (Supplementary material: Table S5), while the treatments *Control* and *Deadwood* did not have significant indicator species (Fig. 4b). Moreover, when comparing the distribution of true bugs between treatments with open and closed canopies, the proportion of individuals for each species was collected predominately in open canopy treatments (i.e., *Gap* and *Gap* + *Deadwood*), and 27 species were significantly associated to open habitats (Fig. 5). In contrast, there were no species with significant association to closed-canopy treatments.

### Open habitats as origins of true bug species in gaps

**Fig. 6:**
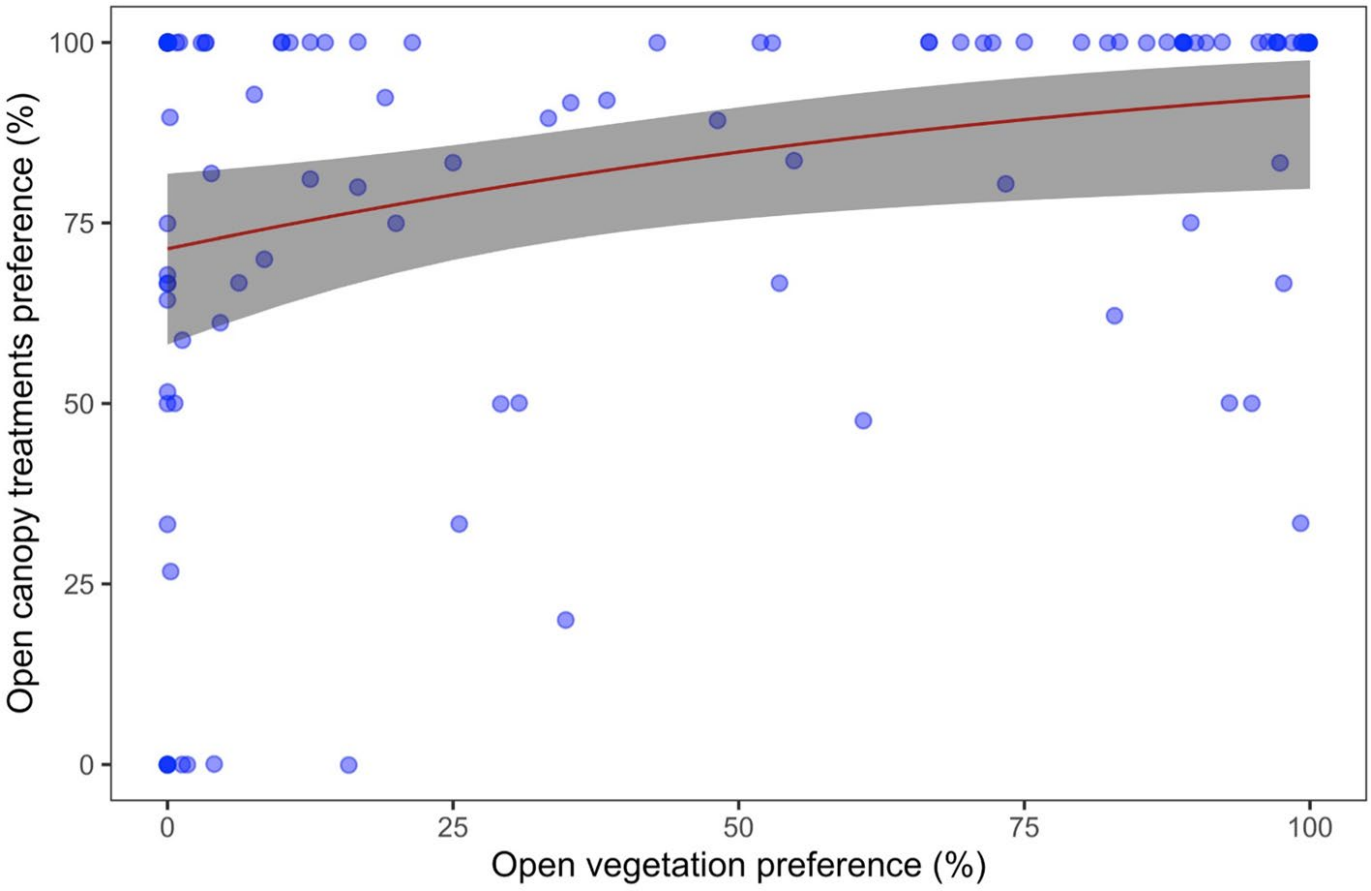
Logistic regression between the open-vegetation preference (calculated based on grassland abundances sampled from 2008 to 2018) versus the open canopy preference (based on abundances found in the treatments *Gap* and *Gap* + *Deadwood*) for 114 species of Heteroptera

The comparison of each species found in this experiment with the monitoring data collected over 11 years (2008–2018) showed that species colonizing open canopy treatments were species that have shown higher preference for sites with open vegetation in the past (*χ*^2^ = 6.037, df = 1, *P* = 0.014) (Fig. 6). Conversely, we did not find evidence that species with high preference for canopies were colonizing the experimental treatments with open canopy (*χ*^2^ < 0.001, df = 1, *P* = 0.978; Supplementary material: Fig. S8). Finally, there was no difference in dispersal ability (CWM) between treatments (*χ*^2^ = 4.156, df = 3, *P* = 0.245), with values for dispersal ability in all four treatments very close to the maximum of 1 (Supplementary material: Fig. S9a). In turn, mean body size was significantly different between treatments (*χ*^2^ = 41.307, df = 3, *P* < 0.001), with *Deadwood* and open canopy treatments promoting communities with larger body sizes compared to the *Control* treatment (Supplementary material: Fig. S9b).

## Discussion

Recently created forest gaps can increase the local abundance and diversity of many organisms due to, e.g., higher light availability, accelerated nutrient cycling, and higher temperatures (Leidinger et al. 2019; Eckerter et al. 2021; Lettenmaier et al. 2022). We used a replicated, full-factorial forest experiment established at nine sites in Germany to determine if (1) gap creation and deadwood availability affect the abundance and the number of a flying insect group, (2) if gaps and closed forests share species composition, and (3) if species colonizing gaps are mostly associated with closed or open habitats. Open conditions within forests increased the number of individuals and species of true bugs compared to closed-canopy treatments. At the community level, there were clear differences in terms of species composition between the open (*Gap* and *Gap* + *Deadwood*) and closed-canopy treatments (*Control* and *Deadwood*). Species colonizing these gaps were often herbivores and species associated with the herb layer. Species colonizing gaps also had on average larger body sizes and a clear preference for open habitats, i.e., the species were usually found in grasslands but not in closed forests. Our results suggest that following gap creation, there is a rapid change in the Heteroptera community structure with an increase in true bug diversity recruited from the surrounding open landscape.

### More species in gaps

Forest gaps promote plant diversity by increasing the occurrence of species adapted to high-light requirements and with high dispersal ability (Degen et al. 2005; Naaf and Wulf 2007). The change in plant communities after gap formation has been shown to affect the animal communities that colonize these newly created environments (Schumann et al. 2003; Laurance 2004; Erasmy et al. 2021). In our study, we found that, in contrast to closed-canopy treatments, gap treatments increased true bug species richness and diversity. This increase was partially mediated by a higher abundance of true bugs (i.e., more-individual hypothesis, Srivastava and Lawton 1998). However, even after controlling for abundance, the higher number of species was maintained and the true bug communities in open canopy treatments were consistently more diverse. Such a pattern may be common for insects in habitats with more light and higher temperatures (Achury et al. 2022; Lettenmaier et al. 2022) and might be related to the metabolic ecology of ectotherms (Prather et al. 2018), where greater quatities of direct radiation in gaps increase the abundance and population growth of insects due to higher temperatures. Concurently, an increased amount and diversity of food resources, available for herbivores (Richards and Windsor 2007), might enhance the higher diversity in gaps. Although we could not directly test if gaps increased plant diversity, several studies have shown that forest gaps promoted the establishment, regeneration, and diversity of vegetation in natural and managed forests (Bauhus and Bartsch 2005; Zhu et al. 2014). These changes in vegetation likely explain our results on food preferences and stratum use, where herbivores and species associated with the herbacious layer were the groups driving the increased diversity in experimental gaps. Thus, for species that were never found in the closed-canopy treatments (e.g., *Harpocera thoracica* and *Megaloceroea recticornis*) both mechanisms, temperature and increased diversity in food resources, may have contributed to immigration and successful establishment of these species.

### Gaps harbored different species composition

All indicator species were significantly associated with the gap treatments, but not with the closed forest or the deadwood treatments, mirroring the results from the community analyses where communities from gap treatments were different from those of the closed forest treatments (e.g., Hägglund et al. 2015). This lack of significant indicator species associated with closed forest treatments (*Control* and *Deadwood* treatments) may be explained due to the fact that the species occurring in these closed treatments were also species frequently found in gap treatments. Moreover, all of the indicator species, with the exception of the omnivore *Dicyphus errans*, were herbivores. This trophic guild showed the greatest increases in both the number of species and individuals, which reflects their prevalence in the number of indicator species compared with the other two guilds, i.e., carnivores and omnivores.

Consistent with other studies evaluating the deadwood effect on insects (Staab et al. 2022), in our experiment, the deadwood treatment did not yet have a strong effect on true bug communities. This finding may be a consequence that true bugs do not feed on deadwood itself and as most of the phytophagous true bug species feed on herbs rather than trees. Nevertheless, there are many predatory species that could potentially feed on other arthropod species feeding on deadwood (Araújo et al. 2007). However, we only evaluated the first 2 years of the experiment when deadwood is in the first stages of decomposition (Edelmann et al. 2023). Other studies have shown clear differences in the insect communities of increasing decomposition stages (Neff et al. 2022; Seibold et al. 2022). Although the presence of deadwood increased the abundance, but not the richness, of true bugs only in the second year after the experiment was established (i.e. 2021), these values are intermediate and do not resemble the communities of true bugs in treatments with an open canopy. Given the role of time on overall pattern of deadwood decay (Herrmann et al. 2015), a temporal analysis combined with the plant dynamics in gaps with vs. without deadwood will in the future elucidate how the presence of deadwood in gaps affects animal community assembly.

### Gaps promoted open land species

When we compared our species communities to those occurring in grasslands outside the forests, we found that species in gaps were recruited from species that preferentially occur in grasslands, indicating that these species rarely, if ever venture into closed forests. Even if they disperse into closed forest, as long as there is no suitable open habitat, they likely either die or disperse further. The true bug species investigated here may be following a pattern of metapopulation dynamics (Price et al. 2011), where forest gaps are colonized by species that prefer open habitats and have to disperse into gaps formed within the closed forest (Seibold et al. 2014). As a caveat, we based our measure on open vs. closed habitat preference on an empirical evaluation that only included grasslands vs. forests (Penone et al. 2019). The advantage is that this is a quantitative measure of preference rather than a literature-based classification of habitat preferences, with the disadvantage that our study did not include other types of open habitats than grasslands, such as croplands, fields, or small tree groups or hedges in the open landscape. In contrast, we did not find evidence that gaps were colonized by species with preference for canopies. Compared to the understory, forest canopies offer a wider range of abiotic and biotic conditions (Müller et al. 2018), which support more species and individuals in some European forests (e.g., Gossner and Ammer 2006). However, most studies in forest (including the present) are restricted to the understory and, therefore, miss information from the canopy that that may provide insights into mechanisms responsible for how animal communities respond to gap formation.

Despite the fact that gap-colonizing species in our study must have dispersed to the gaps, our measure of dispersal ability found no difference in dispersal capacity between gap and closed forest communities. One likely reason is that our measure was relatively coarse, i.e., not based on actual flight capability, measured, e.g., using flight mills (Robinet et al. 2019), but on wing dimorphism. This can reveal patterns of differences in dispersal ability when differences are large (Simons et al. 2016), indicating that the species that form part of these communities are good disperses, but more detailed trait measurements are necessary to deduce finer-scale differences in flight ability, especially because species that do not have good flight abilities are unlikely to be collected in flight-interception traps. For body size, we found that communities inhabiting experimental treatments were on average composed by larger species than in controls. The ability of organisms to disperse is generally positively associated with increasing body size (Dingle et al. 1980; Hirt et al. 2017; Anderson and Fahimipour 2021). In addition, larger species may have an advantage in coping with environmental stressors that are associated with dispersal limitation, such as thermal tolerance (Leiva et al. 2019) and food requirements (Brändle et al. 2000). For instance, in our experiment, the open canopy treatments, that are exposed to higher levels of radiation and have a greater diversity of food resources, may have favored the colonization of larger and more polyphagous hervivores species. Nevertheless, additional research is needed to determine how species reach the gaps, i.e., whether they are flying through the closed forests (and hence are occasionally captured within forests), or above the forest canopy. Managed forests, at least in Central Europe, are characterized by a close network of forest paths to allow machinery to move within the forests. It is possible that, in our experimental set up, this facilitated the colonization of gaps by open land species, as the change in true bug communities occurred rapidly already in the first year. Possibly, the distance that the species had to cross through closed forests was smaller than it would have been in a natural forests without such network of roads.

## Conclusions

Our study supports the notion that animal communities assembling in forest gaps contain a high fraction of species that, in a forest landscape, only or largely occur in gaps (Lehnert et al. 2013). As such, forest gaps contribute significantly to overall forest gamma diversity (Schall et al. 2018). Our study was conducted in the framework of a large-scale field experiment where gap (canopy opening) and deadwood presence was manipulated independently. We found an overwhelming effect of gap on the true bug communities with an effect of deadwood only in gaps. Further studies with species more closely associated with deadwood including saproxylic beetles will show how gap creation interacts with deadwood presence in structuring animal communities in forests.

## Supplementary Information

Below is the link to the electronic supplementary material.

Supplementary file1 (DOCX 1575 KB)

## Data Availability

All raw data are permanently archived and are available in the bExIS repository (https://www.bexis.uni-jena.de/). Accession number 31245 (Hemiptera), 30,938 (vegetation measurements). Climate data are also available from BExIS (https://www.bexis.uni-jena.de/tcd/PublicClimateData/Index).

## Code availability

The statistical code to reproduce the results reported in this study will be available in the BExIS repository (https://www.bexis.uni-jena.de/).
