# Supplementary material for: Forest gaps increase true bug diversity by recruiting open land species
Source: Oecologia. 2023 Jun 4;202(2):299–312. doi: 10.1007/s00442-023-05392-z (PMC10307703; doi:10.1007/s00442-023-05392-z)
Supplement: Supplementary file 1 — Supplementary file1 (DOCX 1575 KB) [file 442_2023_5392_MOESM1_ESM.docx]

**Supplementary material**

**Forest gaps increase true bug diversity by recruiting open land species**

**Rafael Achury^*1^, Michael Staab^2^, Nico Blüthgen^2^, Wolfgang W. Weisser^1^**

^1^ Terrestrial Ecology Research Group, Department of Life Science Systems, School of Life Sciences, Technische Universität München, Freising, Germany

^2^ Ecological Networks Lab, Technische Universität Darmstadt, Darmstadt, Germany

*** Correspondence:** Rafael Achury, Terrestrial Ecology Research Group, Department of Ecology and Ecosystem Management, Technical University of Munich, Freising, 85354, Germany. E-mail: rafael.achury@tum.de

Rafael Achury (0000-0003-0435-3594)

Michael Staab (0000-0003-0894-7576)

Nico Blüthgen (0000-0001-6349-4528)

Wolfgang W. Weisser (0000-0002-2757-8959)

Table S1. True bug species collected during 2020 and 2021 in the four experimental treatments. For each species, the total number of individuals captured per treatment is shown.

| Species | Treatments | | | | Total  abundance |
| --- | --- | --- | --- | --- | --- |
|  | Control | Deadwood | Gap | Gap + Deadwood |  |
| *Acanthosoma haemorrhoidale* |  |  | 2 |  | 2 |
| *Aelia acuminata* |  |  | 8 | 5 | 13 |
| *Amblytylus nasutus* |  |  | 2 | 1 | 3 |
| *Amphiareus obscuriceps* |  |  | 1 |  | 1 |
| *Anthocoris confusus* | 1 | 1 |  | 4 | 6 |
| *Anthocoris nemoralis* | 2 |  | 1 |  | 3 |
| *Anthocoris nemorum* |  | 2 |  |  | 2 |
| *Aphanus rolandri* |  | 1 | 2 | 1 | 4 |
| *Aradus conspicuus* |  | 2 |  | 1 | 3 |
| *Aradus depressus* |  | 9 | 9 | 10 | 28 |
| *Bathysolen nubilus* |  |  |  | 1 | 1 |
| *Berytinus crassipes* |  |  | 1 | 1 | 2 |
| *Berytinus minor* |  |  | 1 | 1 | 2 |
| *Calocoris affinis* |  |  |  | 2 | 2 |
| *Campyloneura virgula* | 5 | 6 | 1 | 3 | 15 |
| *Capsodes gothicus* |  |  |  | 1 | 1 |
| *Capsus ater* |  | 1 | 2 | 3 | 6 |
| *Capsus wagneri* |  |  |  | 1 | 1 |
| *Carpocoris fuscispinus* | 4 | 20 | 68 | 54 | 146 |
| *Carpocoris purpureipennis* | 1 | 9 | 43 | 39 | 92 |
| *Ceraleptus gracilicornis* |  |  | 4 | 9 | 13 |
| *Ceraleptus lividus* |  |  | 1 | 1 | 2 |
| *Chlamydatus pullus* |  | 2 |  | 1 | 3 |
| *Closterotomus biclavatus* |  |  | 1 | 2 | 3 |
| *Closterotomus norwegicus* |  |  | 2 |  | 2 |
| *Coptosoma scutellatum* |  |  | 1 | 1 | 2 |
| *Coreus marginatus* |  | 1 | 6 | 5 | 12 |
| *Coriomeris denticulatus* |  |  | 2 | 2 | 4 |
| *Corizus hyoscyami* |  |  | 3 | 2 | 5 |
| *Cydnus aterrimus* |  | 1 | 1 |  | 2 |
| *Deraeocoris flavilinea* |  |  | 1 |  | 1 |
| *Deraeocoris lutescens* | 10 | 5 | 10 | 6 | 31 |
| *Deraeocoris ruber* |  |  | 1 | 2 | 3 |
| *Dicyphus errans* |  |  | 4 | 2 | 6 |
| *Dicyphus pallidus* |  |  |  | 1 | 1 |
| *Dicyphus stachydis* |  |  | 4 | 1 | 5 |
| *Dolycoris baccarum* | 11 | 61 | 155 | 154 | 381 |
| *Drymus ryeii* | 1 | 1 |  |  | 2 |
| *Drymus sylvaticus* |  | 3 | 6 |  | 9 |
| *Dryophilocoris flavoquadrimaculatus* |  |  |  | 1 | 1 |
| *Emblethis verbasci* |  |  |  | 1 | 1 |
| *Eurydema oleracea* |  | 6 | 11 | 19 | 36 |
| *Eurydema ornata* |  | 2 | 5 | 4 | 11 |
| *Eurygaster testudinaria* |  |  | 2 |  | 2 |
| *Eysarcoris venustissimus* |  | 12 | 22 | 2 | 36 |
| *Gastrodes abietum* |  |  | 1 |  | 1 |
| *Gonocerus acuteangulatus* |  |  | 1 |  | 1 |
| *Graphosoma lineatum* |  |  |  | 1 | 1 |
| *Halticus apterus* |  |  | 1 |  | 1 |
| *Harpocera thoracica* |  |  | 6 | 30 | 36 |
| *Himacerus apterus* |  |  |  | 2 | 2 |
| *Himacerus mirmicoides* |  |  | 1 |  | 1 |
| *Kalama tricornis* |  |  |  | 1 | 1 |
| *Kleidocerys resedae* |  | 7 | 6 | 5 | 18 |
| *Leptoglossus occidentalis* |  |  |  | 1 | 1 |
| *Leptopterna dolabrata* |  |  | 3 | 1 | 4 |
| *Liocoris tripustulatus* |  | 2 | 12 | 11 | 25 |
| *Loricula elegantula* | 1 |  |  |  | 1 |
| *Lygocoris pabulinus* |  | 2 |  | 4 | 6 |
| *Lygus gemellatus* |  |  |  | 1 | 1 |
| *Lygus pratensis* | 5 | 26 | 33 | 18 | 82 |
| *Lygus rugulipennis* | 1 | 1 | 1 | 1 | 4 |
| *Megaloceroea recticornis* |  |  | 5 | 10 | 15 |
| *Megalonotus chiragra* |  | 1 |  | 3 | 4 |
| *Megalonotus sabulicola* |  |  | 1 |  | 1 |
| *Mermitelocerus schmidtii* |  | 5 | 4 | 5 | 14 |
| *Metatropis rufescens* |  |  |  | 1 | 1 |
| *Metopoplax ditomoides* |  |  | 1 | 2 | 3 |
| *Miris striatus* |  |  | 1 | 1 | 2 |
| *Nabis flavomarginatus* |  |  | 1 | 1 | 2 |
| *Nabis pseudoferus* | 4 | 7 | 3 | 7 | 21 |
| *Notostira elongata* |  |  | 1 |  | 1 |
| *Orius horvathi* |  |  | 2 | 3 | 5 |
| *Orius minutus* |  | 4 |  | 1 | 5 |
| *Orius vicinus* |  | 1 | 1 | 2 | 4 |
| *Orthocephalus coriaceus* |  |  | 1 |  | 1 |
| *Orthocephalus saltator* |  |  | 1 |  | 1 |
| *Orthops basalis* |  | 1 |  | 2 | 3 |
| *Orthops campestris* |  |  |  | 1 | 1 |
| *Orthotylus prasinus* | 1 |  |  |  | 1 |
| *Oxycarenus lavaterae* |  |  | 1 |  | 1 |
| *Palomena prasina* | 9 | 74 | 394 | 326 | 803 |
| *Palomena viridissima* |  |  | 4 | 2 | 6 |
| *Peribalus strictus* |  | 11 | 61 | 81 | 153 |
| *Peritrechus lundii* |  |  |  | 1 | 1 |
| *Peritrechus nubilus* |  |  |  | 1 | 1 |
| *Phymata crassipes* |  |  | 1 |  | 1 |
| *Piesma maculatum* |  |  | 1 |  | 1 |
| *Piezodorus lituratus* | 2 | 1 | 4 | 3 | 10 |
| *Pinalitus cervinus* |  |  | 1 | 2 | 3 |
| *Plagiognathus arbustorum* |  |  | 5 | 1 | 6 |
| *Plagiognathus chrysanthemi* |  |  | 1 |  | 1 |
| *Plagiognathus vitellinus* |  | 1 |  |  | 1 |
| *Psallus albicinctus* |  | 1 |  |  | 1 |
| *Psallus mollis* | 1 |  |  |  | 1 |
| *Psallus punctulatus* |  |  |  | 1 | 1 |
| *Psallus varians* | 54 | 51 | 82 | 68 | 255 |
| *Raglius alboacuminatus* |  |  | 2 |  | 2 |
| *Rhabdomiris striatellus* |  | 1 |  | 1 | 2 |
| *Rhopalus parumpunctatus* |  |  | 1 |  | 1 |
| *Rhopalus subrufus* |  | 2 | 13 | 4 | 19 |
| *Rhyparochromus vulgaris* |  |  | 4 | 2 | 6 |
| *Sciocoris homalonotus* | 1 |  | 1 | 3 | 5 |
| *Scoloposcelis pulchella* |  |  |  | 1 | 1 |
| *Scolopostethus grandis* |  |  | 1 |  | 1 |
| *Scolopostethus thomsoni* | 1 |  |  | 1 | 2 |
| *Sehirus luctuosus* |  | 1 | 6 | 6 | 13 |
| *Stenodema laevigata* | 2 | 9 | 19 | 26 | 56 |
| *Stenotus binotatus* |  |  | 1 | 1 | 2 |
| *Stictopleurus abutilon* |  | 1 |  | 1 | 2 |
| *Syromastus rhombeus* |  |  |  | 1 | 1 |
| *Temnostethus pusillus* | 2 |  |  |  | 2 |
| *Tingis pilosa* |  |  | 1 |  | 1 |
| *Trapezonotus dispar* |  |  | 7 | 2 | 9 |
| *Tritomegas bicolor* | 1 |  |  | 1 | 2 |
| *Troilus luridus* |  |  |  | 4 | 4 |
| *Tropistethus holosericus* |  |  | 1 |  | 1 |
| Total Abundance | 120 | 355 | 1082 | 999 | 2556 |

Table S2. Pairwise contrasts using Bonferroni adjustments for a) the number of species per plot and b) the number of individuals between each pair of experimental treatments. Significant contrasts (*P* < 0.05) are in bold.

|  | Estimate (SE) | t-ratio | P-value |
| --- | --- | --- | --- |
| a) *Richness contrasts* |  |  |  |
| Control – Deadwood | -0.573 (0.206) | -2.784 | 0.055 |
| **Control – Gap** | **-1.199 (0.194)** | **-6.164** | **< 0.001** |
| **Control – Gap + Deadwood** | **-1.256 (0.194)** | **-6.484** | **< 0.001** |
| **Deadwood – Gap** | **-0.625 (0.172)** | **-3.632** | **0.006** |
| **Deadwood – Gap + Deadwood** | **-0.683 (0.173)** | **-3.959** | **0.003** |
| Gap – Gap + Deadwood | -0.058 (0.159) | -0.363 | 1.000 |
| b) *Abundance contrasts* |  |  |  |
| Control – Deadwood | -0.838 (0.305) | -2.747 | 0.060 |
| **Control – Gap** | **-1.9274 (0.303)** | **-6.365** | **< 0.001** |
| **Control – Gap + Deadwood** | **-1.9633 (0.297)** | **-6.604** | **< 0.001** |
| **Deadwood – Gap** | **-1.0893 (0.283)** | **-3.848** | **0.003** |
| **Deadwood – Gap + Deadwood** | **-1.1251 (0.290)** | **-3.879** | **0.003** |
| Gap – Gap + Deadwood | -0.0359 (0.281) | -0.127 | 1.000 |

Table S3. Pairwise contrasts using Bonferroni adjustments for the number of species of Heteroptera after accounting for abundance between each pair of experimental treatments. Significant contrasts (*P* < 0.05) are in bold.

|  | Estimate (SE) | z-ratio | P-value |
| --- | --- | --- | --- |
| *Richness contrasts controlled for abundance* |  |  |  |
| **Control – Deadwood** | **-0.467 (0.166)** | **-2.812** | **0.052** |
| **Control – Gap** | **-0.685 (0.176)** | **-3.905** | **0.003** |
| **Control – Gap + Deadwood** | **-0.795 (0.168)** | **-4.726** | **< 0.001** |
| Deadwood – Gap | -0.218 (0.140) | -1.559 | 0.779 |
| Deadwood – Gap + Deadwood | -0.328 (0.133) | -2.456 | 0.122 |
| Gap – Gap + Deadwood | -0.110 (0.103) | -1.064 | 1.000 |

Table S4. Multilevel pairwise comparison (based on PERMANOVA analysis) assessing the dissimilarity for species composition of Heteroptera with Bonferroni adjustment between each pair of experimental treatments. Significant contrasts (*P* < 0.05) are in bold.

| Species composition contrasts | *F* | *P-value* |
| --- | --- | --- |
| Control – Deadwood | 1.89 | 0.306 |
| **Control – Gap** | **6.78** | **0.006** |
| **Control – Gap + Deadwood** | **6.45** | **0.012** |
| Deadwood – Gap | 2.41 | 0.192 |
| Deadwood – Gap + Deadwood | 2.25 | 0.228 |
| Gap – Gap + Deadwood | 0.55 | 1.000 |

Table S5. Results from the indicator species analysis (significance based on Monte Carlo tests) for the association between true bug species and the combination of experimental treatments. Indicator values ranging from 0 (no indication) to 1 (perfect indication). Only significant indicator species are shown (*P* < 0.05). Species are sorted by indicator value, with most strongly associated species shown first.

| Species | Indicator value | *P* | Treatment |
| --- | --- | --- | --- |
| *Megaloceroea recticornis* | 0.577 | < 0.001 | GAP and GAP + Deadwood |
| *Aelia acuminata* | 0.537 | 0.001 | GAP and GAP + Deadwood |
| *Peribalus strictus* | 0.513 | < 0.001 | GAP and GAP + Deadwood |
| *Stenodema laevigata* | 0.474 | 0.003 | GAP and GAP + Deadwood |
| *Palomena prasina* | 0.470 | 0.001 | GAP and GAP + Deadwood |
| *Carpocoris fuscispinus* | 0.470 | 0.004 | GAP and GAP + Deadwood |
| *Trapezonotus dispar* | 0.457 | 0.008 | GAP and GAP + Deadwood |
| *Dolycoris baccarum* | 0.438 | 0.004 | GAP and GAP + Deadwood |
| *Liocoris tripustulatus* | 0.432 | 0.005 | GAP and GAP + Deadwood |
| *Ceraleptus gracilicornis* | 0.407 | 0.009 | GAP and GAP + Deadwood |
| *Sehirus luctuosus* | 0.407 | 0.015 | GAP and GAP + Deadwood |
| *Carpocoris purpureipennis* | 0.405 | 0.008 | GAP and GAP + Deadwood |
| *Dicyphus errans* | 0.378 | 0.044 | GAP and GAP + Deadwood |
| *Palomena viridissima* | 0.378 | 0.044 | GAP and GAP + Deadwood |
| *Rhyparochromus vulgaris* | 0.378 | 0.047 | GAP and GAP + Deadwood |
| *Eurydema oleracea* | 0.346 | 0.047 | GAP and GAP + Deadwood |
| *Rhopalus subrufus* | 0.326 | 0.032 | GAP and GAP + Deadwood |

**
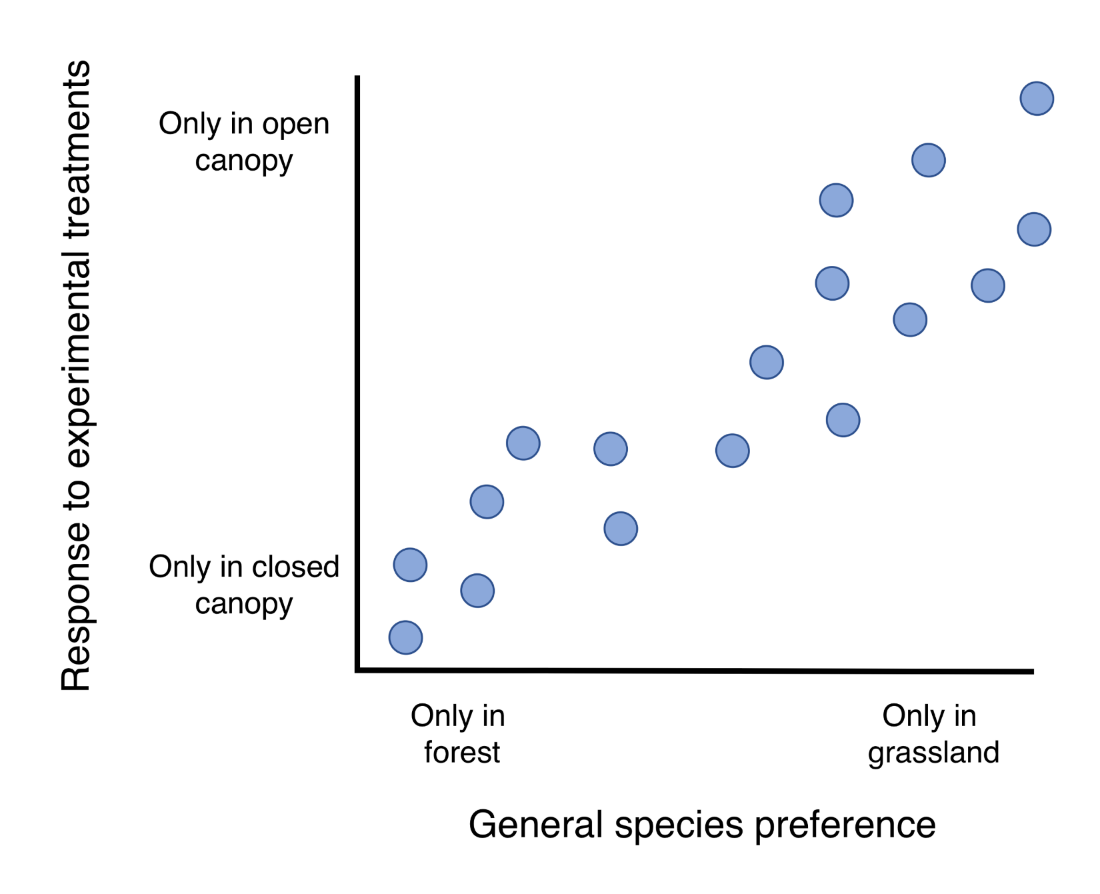
**

Figure S1. Conceptual framework of how the colonization of true bug species might be affected by the general preference of such species for closed (forests) vs. open vegetation (grasslands). Thus, there will be a positive slope if the species found in the open canopy experimental treatments are those that are also found inhabiting open habitats, in this case grasslands.


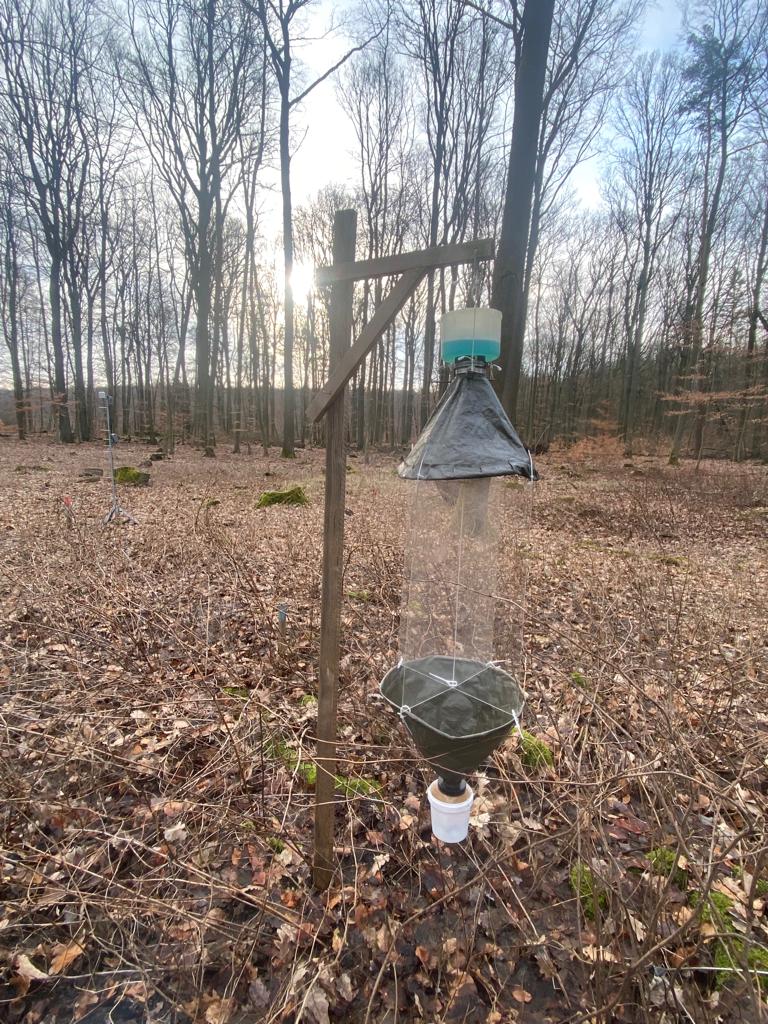


Figure S2. Window trap installed on a wooden frame in one of the treatments (*Gap*) in the Hainich-Dün region. Two transparent crossed windows serve as flight barrier. The containers located at the top and bottom of the funnels are filled with a solution of CuSO4 (3%) and a drop of detergent.

| a) | 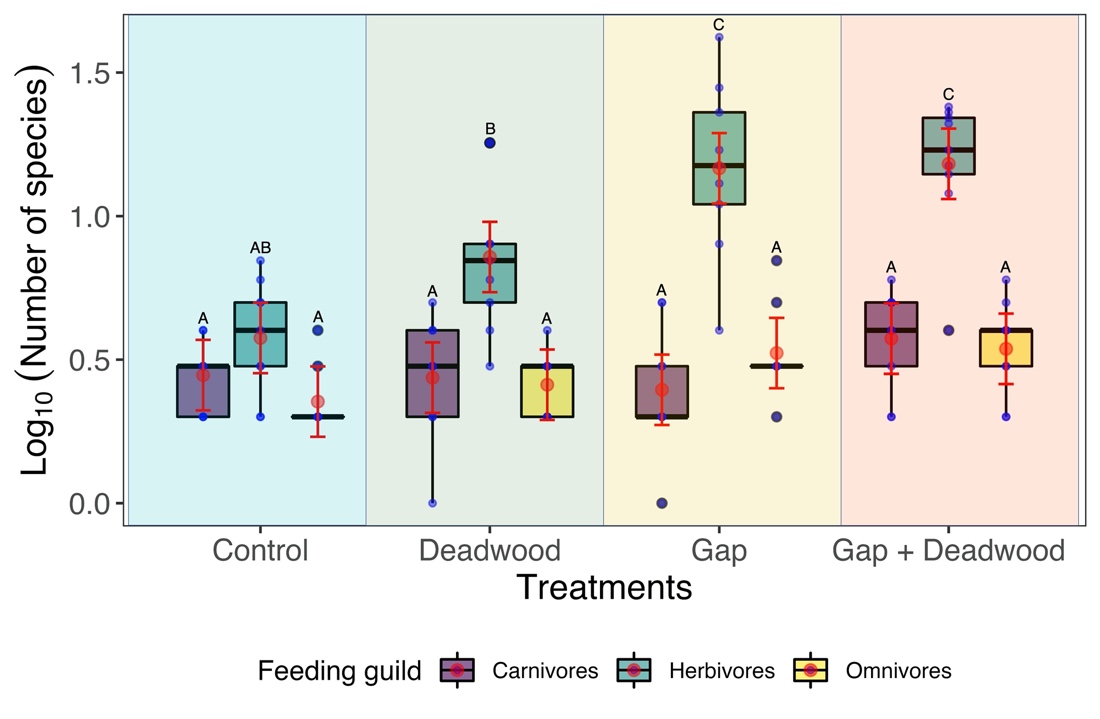 |
| --- | --- |
| b) | 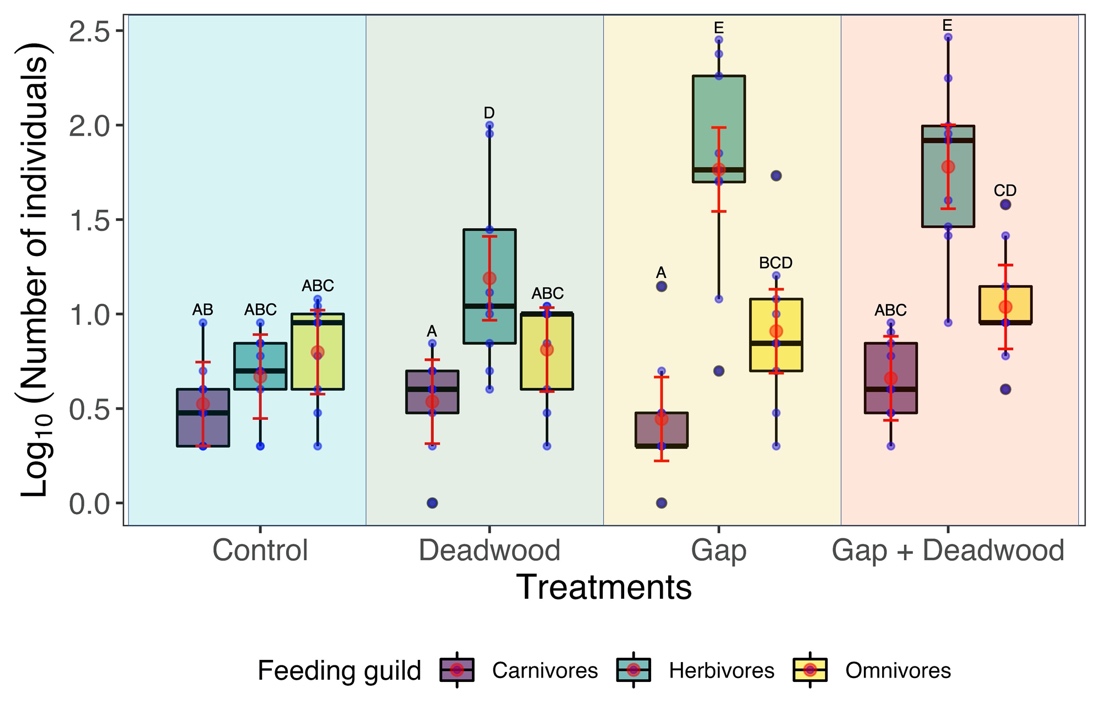 |

Figure S3. Differences in a) number of species and b) individuals per plot among experimental treatments in relation with feeding guild. The interaction between treatments and feeding guilds was significant for number of species (χ^2^ = 18.716; df = 6; *P* = 0.005) and individuals (χ^2^ = 47.237; df = 6; *P* < 0.001). Blue dots are raw data while the red dot is the average estimate by the model (± SE). Different letters indicate statistical significance with pairwise contrast and Bonferroni correction at *P* < 0.05. Models were run with negative binomial error but the y-axes are log transformed for better readability. Background colors represent the color used for each of the treatments in the main text.

| a) | 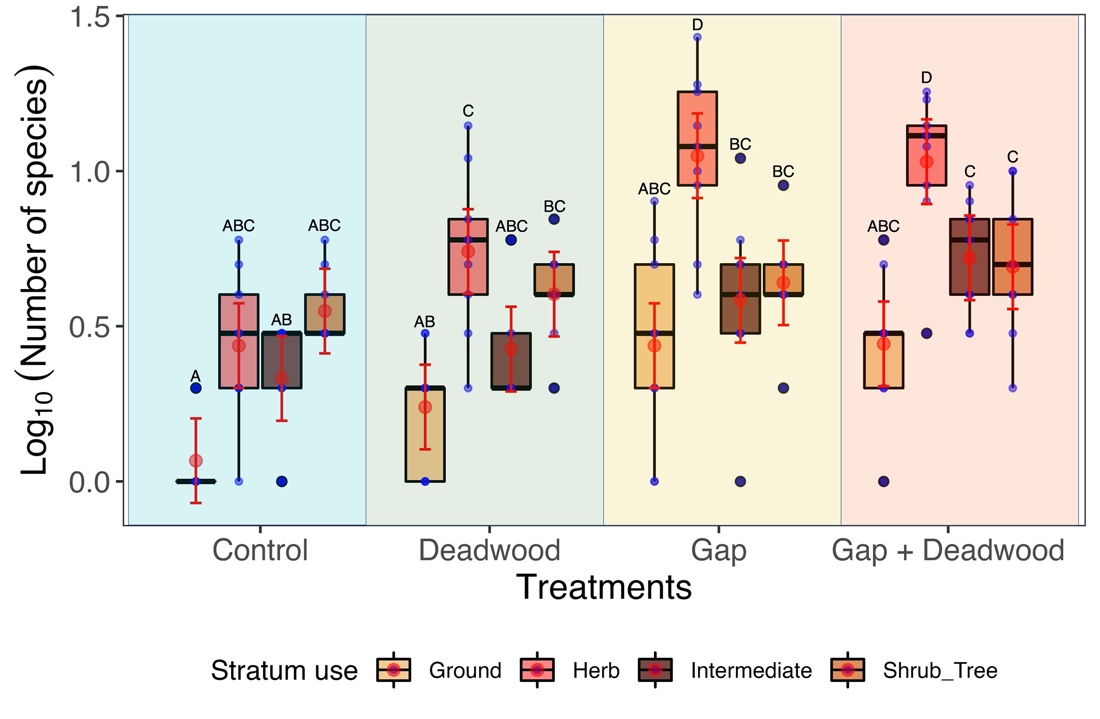 |
| --- | --- |
| b) | 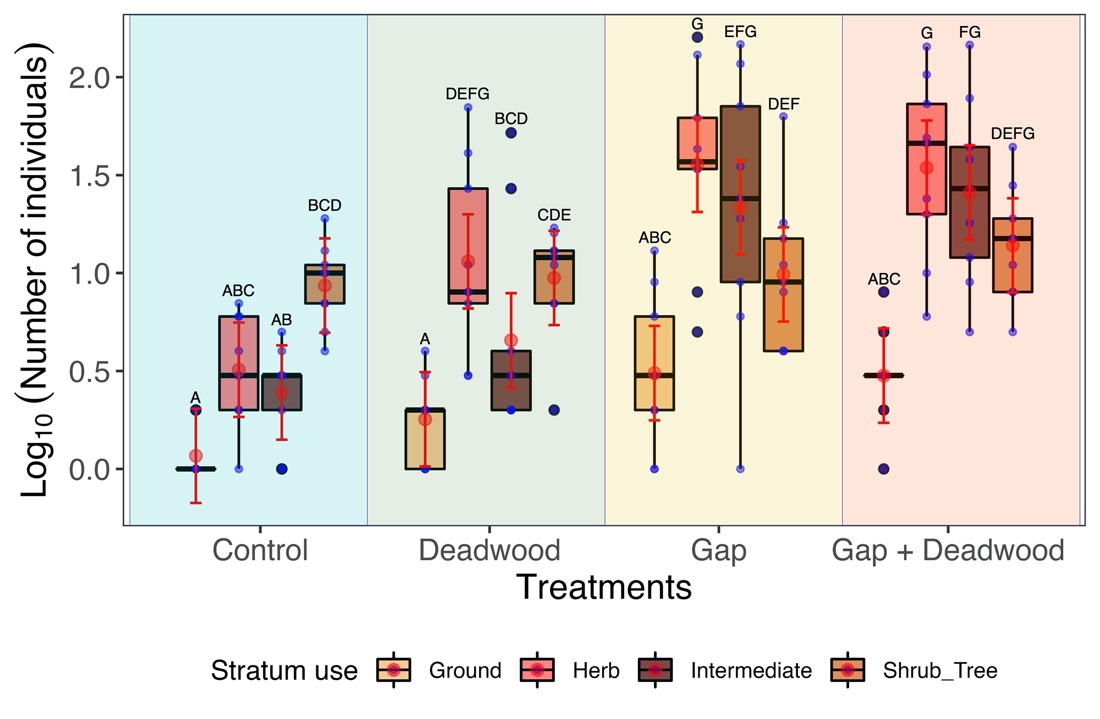 |

Figure S4. Differences in a) number of species and b) individuals per plot among experimental treatments in relation with stratum use. The interaction between treatments and stratum use was significant for number of species (χ^2^ = 19.855; df = 9; *P* = 0.019) and individuals (χ^2^ = 37.086; df = 9; *P* < 0.001). Blue dots are raw data while the red dot is the average estimate by the model (± SE). Different letters indicate statistical significance with pairwise contrast and Bonferroni correction at *P* < 0.05. Models were run with negative binomial error but the y-axes are log transformed for better readability. Background colors represent the color used for each of the treatments in the main text.


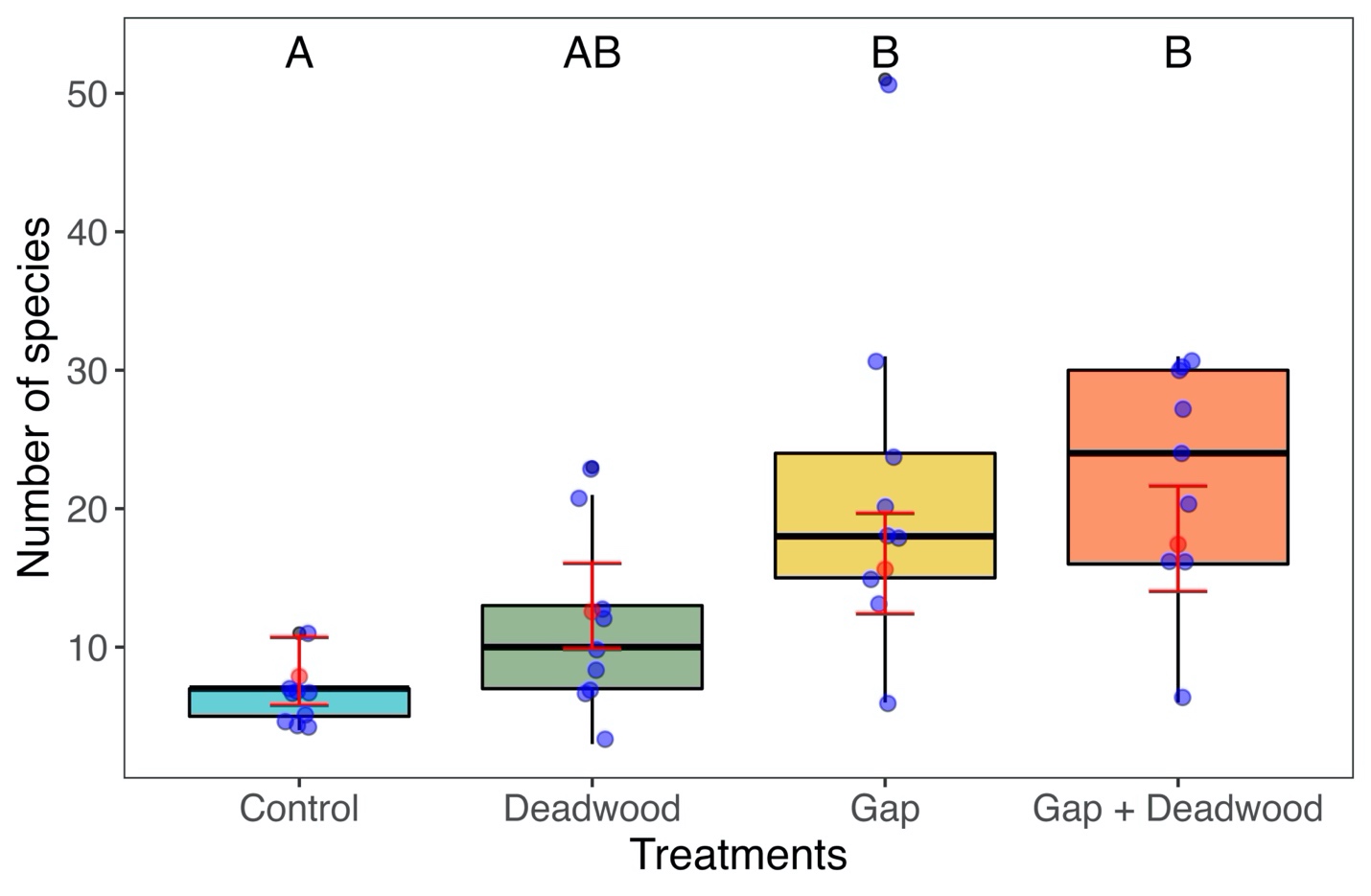
Figure S5. Differences in the number of individuals per plot among experimental treatments when abundance is accounted for. Blue dots are raw data while the red dots give the average estimate by the model (± SE). Different letters indicate statistical significance at *P* < 0.05. Pairwise contrasts are reported in Table S3.


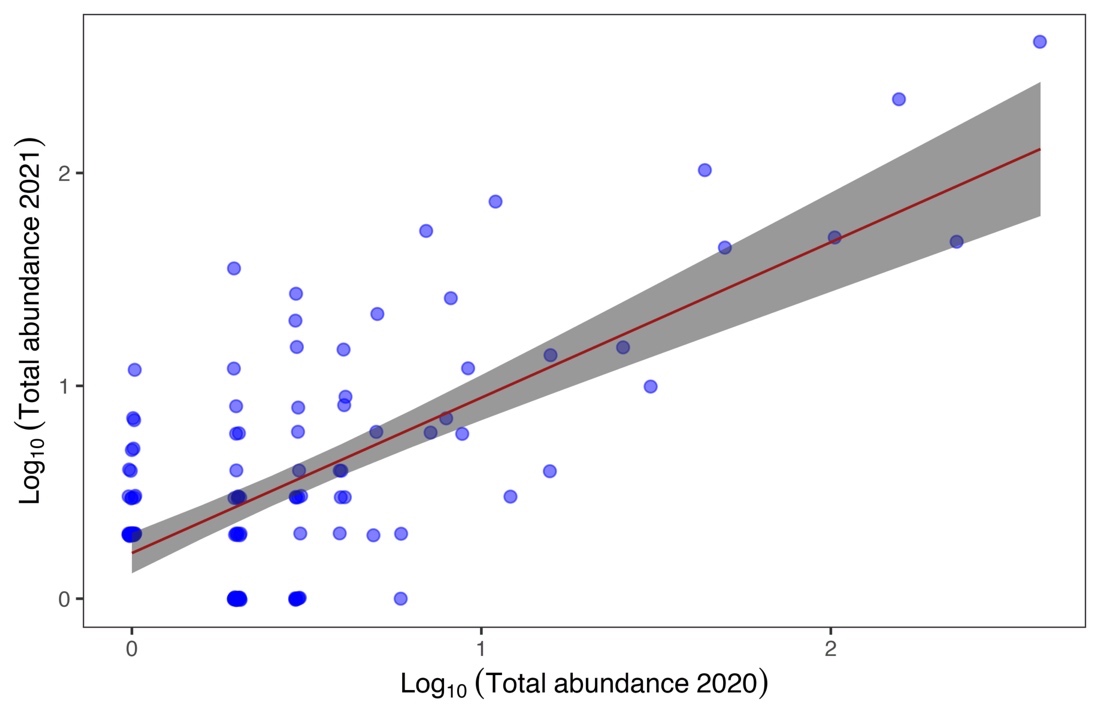


Figure S6. Relationship between the number of individuals per species captured in 2020 and 2021. Pearson’s *r* = 0.68; *t_(120)_* = 10.043, ; *P* < 0.001.

| a) | 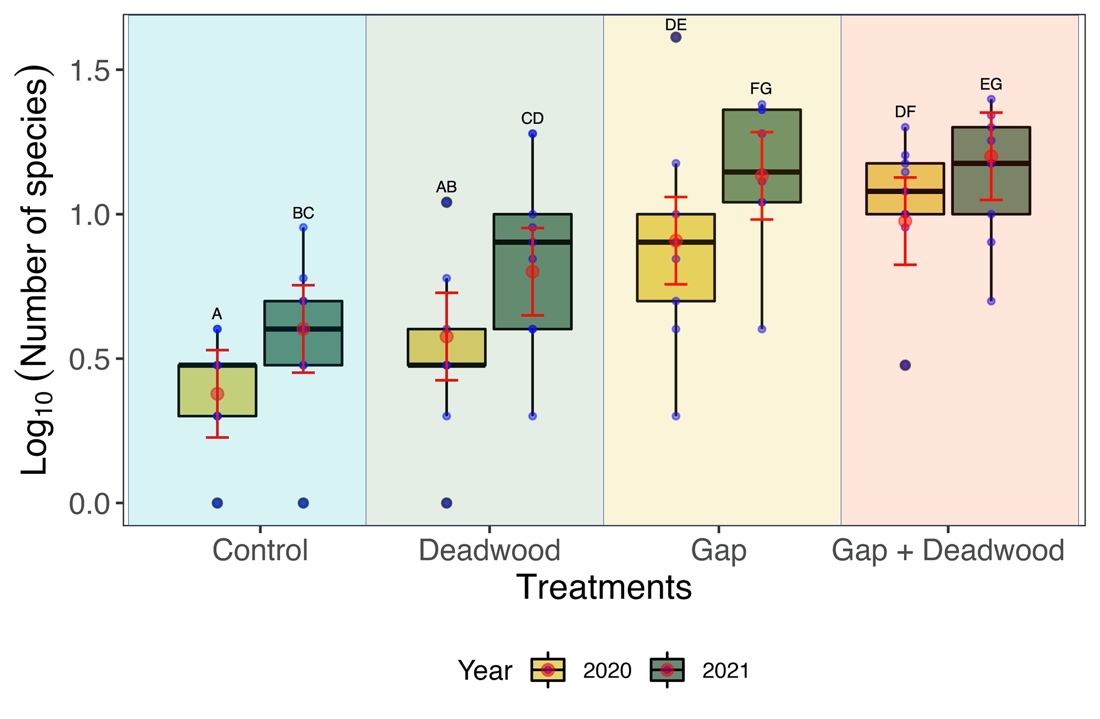 |
| --- | --- |
| b) | 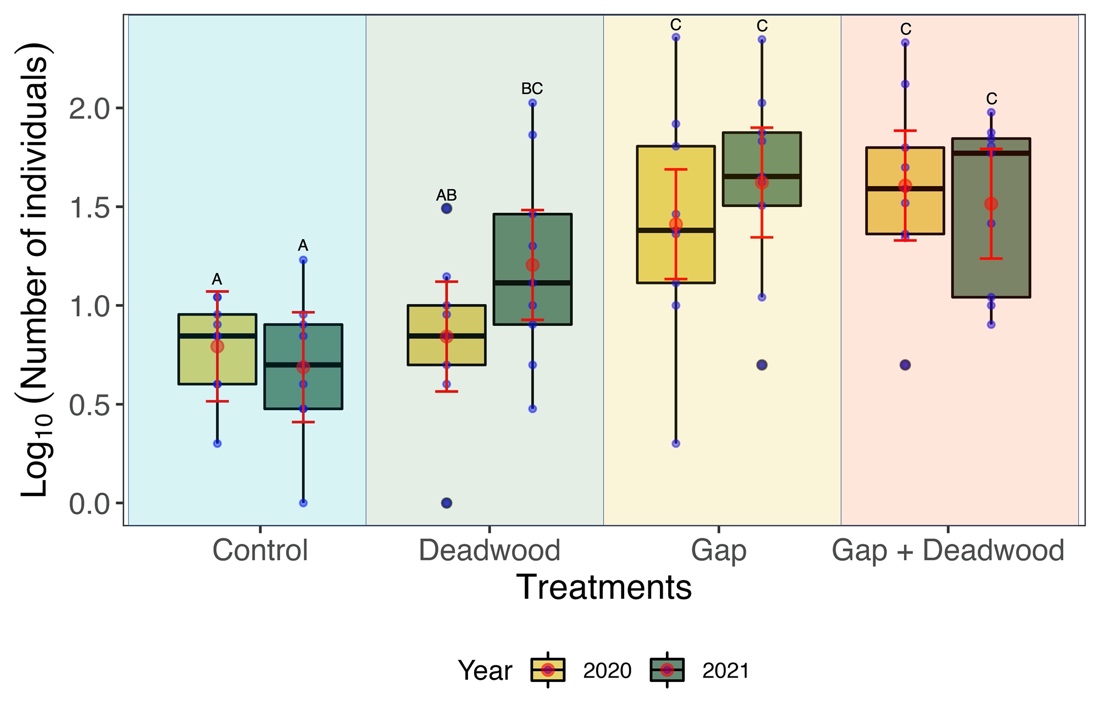 |

Figure S7. Differences in a) number of species and b) individuals per plot among experimental treatments in relation with the year of sampling. Regarding number of species, for the first year of sampling there was no interaction between treatments and years, and the two factors independently were significant (treatment: χ^2^ = 81.178; df = 3; *P* < 0.001, and year: χ^2^ = 15.699; df = 1; *P* < 0.001). In contrast, for number of individuals the interaction between factors was significant (χ^2^ = 8.424; df = 3; *P* = 0.038). Blue dots are raw data while the red dot is the average estimate by the model (± SE). Different letters indicate statistical significance with pairwise contrast and Bonferroni correction at *P* < 0.05. Models were run with negative binomial error but the y-axes are log transformed for better readability. Background colors represent the color used for each of the treatments in the main text.

| 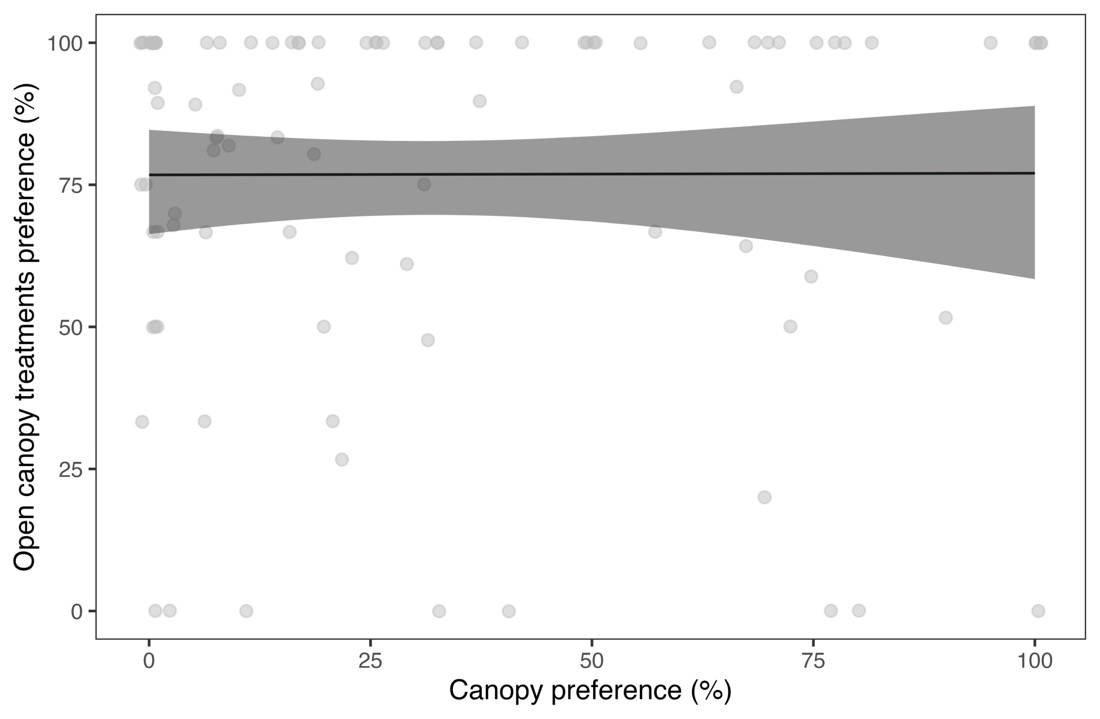 |
| --- |

Figure S8. Logistic regression between the canopy preference (calculated based on canopy abundances sampled from 2008 to 2012) versus the open canopy preference (based on abundances found in the treatments *Gap* and *Gap + Deadwood*).

| a) | 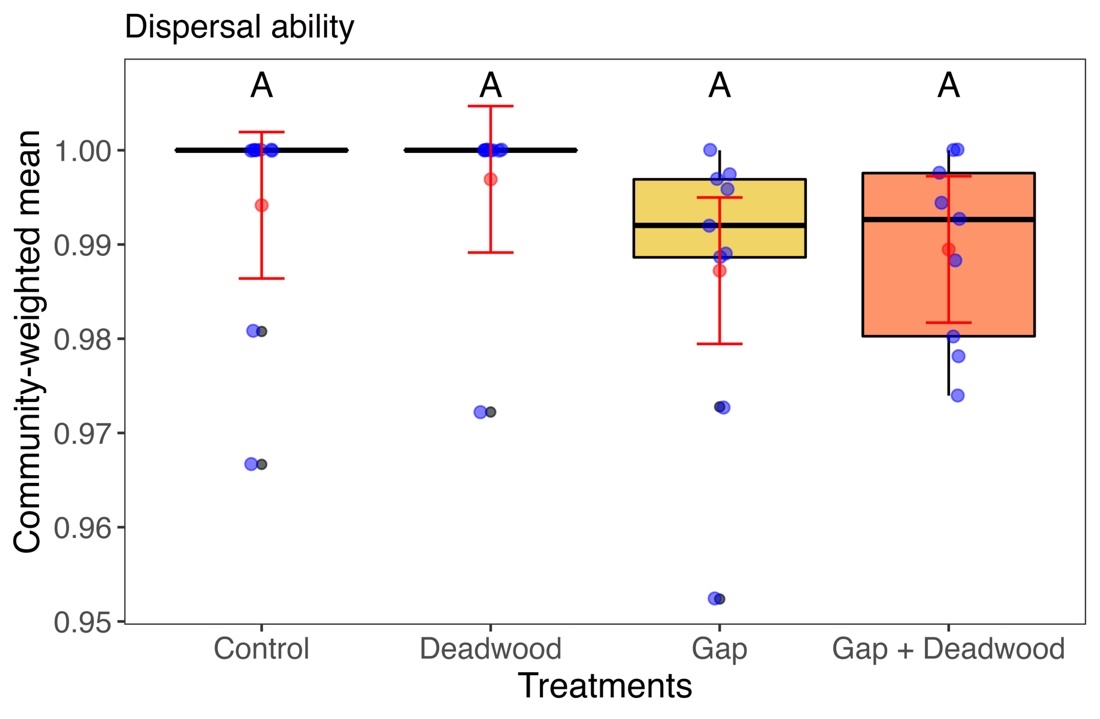 |
| --- | --- |
| b) | 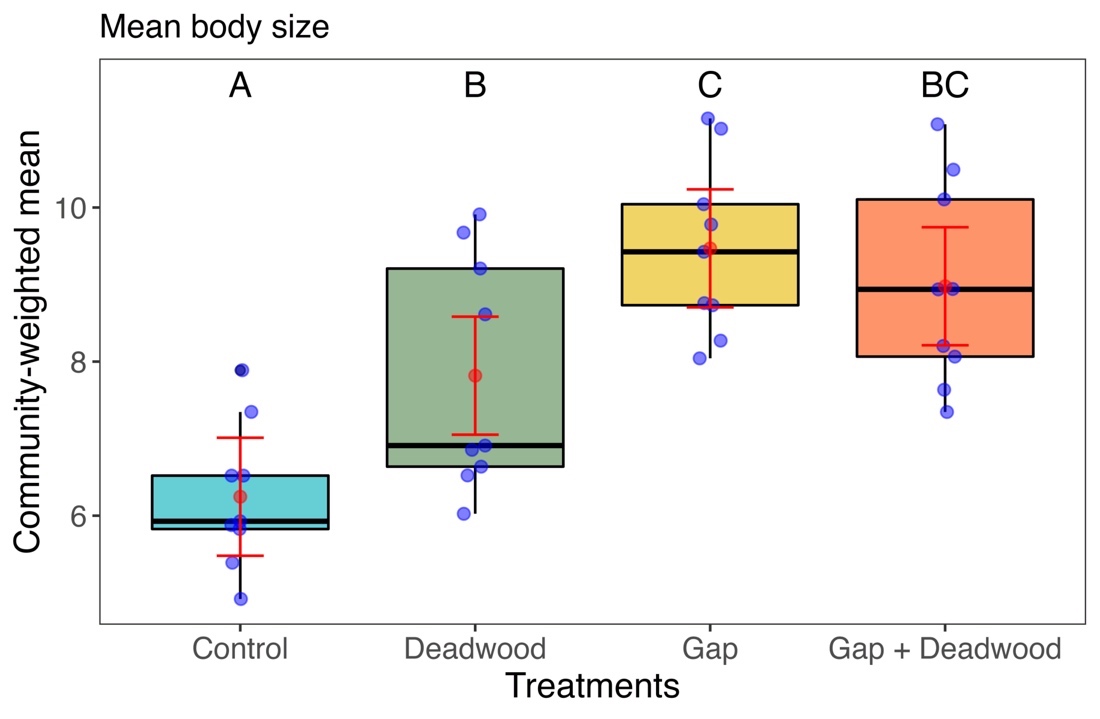 |

Figure S9. Response of the dispersal ability a) and mean body size b) for true bug species defined as community weighted-mean (CMW) to the effect of experimental treatments in forests of the Haninich-Dün region during 2020 and 2021. Blue dots are raw data while the red dot is the average estimate by the model (± SE). Different letters indicate statistical significance with pairwise contrast and Bonferroni correction at *P* < 0.05.
